# Sarecycline inhibits protein translation in *Cutibacterium acnes* 70S ribosome using a two-site mechanism

**DOI:** 10.1093/nar/gkad103

**Published:** 2023-03-02

**Authors:** Ivan B Lomakin, Swapnil C Devarkar, Shivali Patel, Ayman Grada, Christopher G Bunick

**Affiliations:** Department of Dermatology, Yale University School of Medicine, New Haven, CT06520, USA; Department of Molecular Biophysics and Biochemistry, Yale University, New Haven, CT06520, USA; Department of Molecular Biophysics and Biochemistry, Yale University, New Haven, CT06520, USA; Department of Dermatology, Case Western Reserve University School of Medicine, Cleveland, OH 44106, USA; Department of Dermatology, Yale University School of Medicine, New Haven, CT06520, USA; Department of Molecular Biophysics and Biochemistry, Yale University, New Haven, CT06520, USA; Program in Translational Biomedicine, Yale University School of Medicine, New Haven, CT 06520, USA

## Abstract

Acne vulgaris is a chronic disfiguring skin disease affecting ∼1 billion people worldwide, often having persistent negative effects on physical and mental health. The Gram-positive anaerobe, *Cutibacterium acnes* is implicated in acne pathogenesis and is, therefore, a main target for antibiotic-based acne therapy. We determined a 2.8-Å resolution structure of the 70S ribosome of *Cutibacterium acnes* by cryogenic electron microscopy and discovered that sarecycline, a narrow-spectrum antibiotic against *Cutibacterium acnes*, may inhibit two active sites of this bacterium's ribosome in contrast to the one site detected previously on the model ribosome of *Thermus thermophilus*. Apart from the canonical binding site at the mRNA decoding center, the second binding site for sarecycline exists at the nascent peptide exit tunnel, reminiscent of the macrolides class of antibiotics. The structure also revealed *Cutibacterium acnes*-specific features of the ribosomal RNA and proteins. Unlike the ribosome of the Gram-negative bacterium *Escherichia coli*, *Cutibacterium acnes* ribosome has two additional proteins, bS22 and bL37, which are also present in the ribosomes of *Mycobacterium smegmatis and Mycobacterium tuberculosis*. We show that bS22 and bL37 have antimicrobial properties and may be involved in maintaining the healthy homeostasis of the human skin microbiome.

## Graphical Abstract

**Graphical Abstract:**
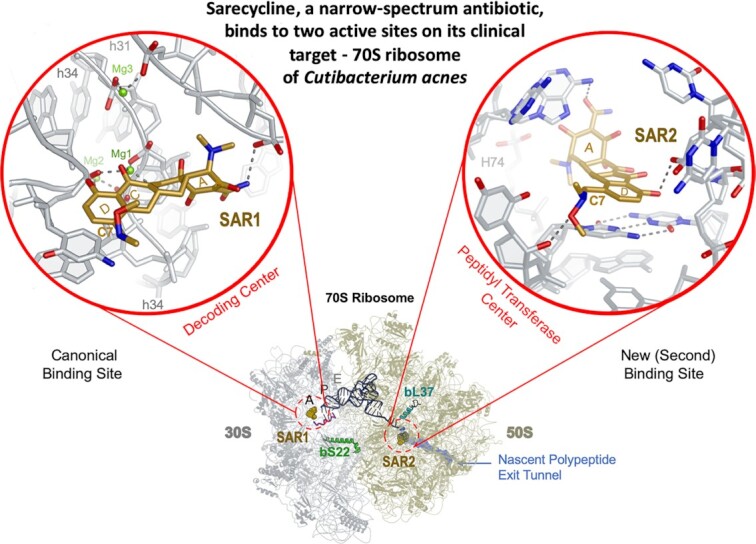
Cutibacterium acnes is the skin commensal implicated in Acne vulgaris pathogenesis. The structure of its ribosome with sarecycline shows a unique mechanism: sarecycline binds two active centers of the ribosome.

## INTRODUCTION

The bacterial ribosome is one of the major therapeutic targets for treatment of human infections and diseases (1). Because ribosomal RNA (rRNA) has highly conserved sequence and structure, many antibiotics targeting the ribosome are active against most eubacteria, providing broad-spectrum activity. While such activity inhibits growth of the target pathogen, it may cause unintended negative effects on the gut and skin microbiota, harming the patient's health and quality of life (2,3). In addition, misuse and overuse of antibiotics promotes antimicrobial resistance (AMR), a major global public health threat facing humanity (4). The development of narrow-spectrum antibacterial drugs that are more pathogen-specific can help to avoid the disruption of the human microbiome and minimize AMR emergence (5). However, precise knowledge of the biochemical and structural basis governing narrow-spectrum activity is needed to enhance antibiotic innovation.

*Cutibacterium acnes* (*C. acnes*) is an anaerobic Gram-positive bacterium and one of the most common and abundant bacterial species present on the human skin as part of the normal flora (2). It predominates in sebaceous areas and plays an important role in pathogenesis of acne vulgaris, and also in implant-associated infections, chronic blepharitis, and endophthalmitis (6–8). Acne mostly affects the skin of the face, neck, upper chest, shoulders and back with non-inflammatory and/or inflammatory lesions, seborrhea, and varied degrees of scarring. Acne affects ∼9.4% of the world population, including around 85% of teenagers and young adults. There are >3 million new cases per year in the United States alone. Despite acne being the eighth most prevalent disease in the world, our molecular understanding of *C. acnes* function is limited (9–12).

**Figure 1.**
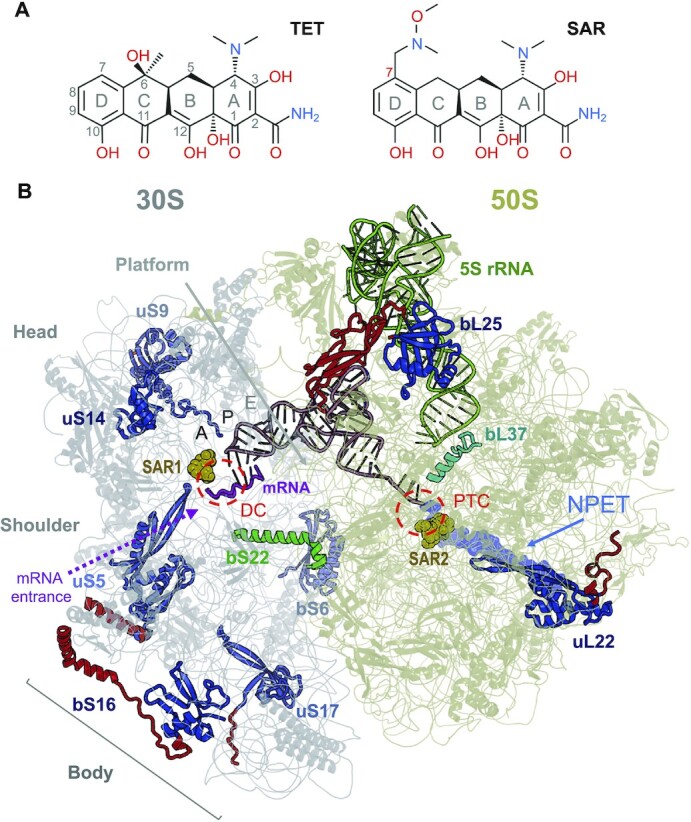
Interaction of Sarecycline with the 70S ribosome from *C. acnes*. (**A**) Chemical structures of TET and SAR. (**B**) Structure of SAR (gold) in complex with the 70S ribosome (30S subunit in gray, 50S subunit in khaki), mRNA (magenta), and P-site tRNA (brown). DC, decoding center; PTC, peptidyl-transferase center; NPET, nascent peptide exit tunnel. *C. acnes-*specific ribosomal proteins are shown in green (bS22) and cyan (bL37), as well as in blue (conserved domains) and red (*C. acnes-*specific extensions).

Sequence analysis and structural data on ribosomes reveal that despite the high degree of conservation, there can be extensive structural differences between distinct species within eubacteria (13–15). These species-specific regions of the ribosome are therefore good targets for screening of new, pathogen-focused inhibitors. Sarecycline (SAR), a third-generation tetracycline-class antibiotic with narrow-spectrum activity toward some gram-positive bacteria, including *C. acnes*, was developed for acne treatment. It has a low propensity for inducing resistance in *C. acnes* (Figure 1A, Supplementary Figure S1) (16). However, it is not clear which structural features of the antibiotic and/or ribosome are responsible for that. All tetracyclines bind between the head and the shoulder of the small ribosomal subunit within the decoding center, thereby interfering with interactions between the anticodon loop of incoming A-site tRNAs and A-site codon of mRNAs (17,18). Tetracycline binding may impair dissociation of translation initiation factor 1 from the 30S preinitiation complex, delaying formation of 70S initiation complexes and slowing onset of the elongation phase of protein synthesis (19). This primary binding site, which we denote the ‘canonical binding site’ (CBS), is located in the pocket formed by helices 31 (h31) and 34 (h34) of the 16S rRNA (20–23). The CBS is the only tetracycline binding site supported by all the available structural data. Other reported sites are likely artifacts caused by misinterpretation of low-resolution crystallographic data or are lower occupancy secondary binding sites, observed only in the 30S subunit. In all recent high-resolution structures of bacterial ribosomes with tetracyclines bound, they bound only the CBS (20,23,24). Importantly, the studies described used ribosomes purified from model Gram-negative organisms *Escherichia coli* (*E. coli*) or *Thermus thermophilus* (*T. thermophilus*). Recently, in the structures of 70S ribosome from Gram-negative pathogen *Acinetobacter baumannii* with tigecycline (TIG) or eravacycline (ERA) bound, in addition to the CBS, a secondary binding site was observed in the central protuberance (for TIG) or in the vicinity of H89 (for ERA) of the 50S subunit. Three TIG molecules stacked on each other in a cavity between 23S rRNA, 5S rRNA and ribosomal protein bL27; ERA dimer interacts with H40 and H89, however, no functional conclusions were reached about these secondary binding sites (24,25).

We showed recently that SAR’s unique 7-[[methoxy(methyl)amino]methyl] group attached to carbon 7 (ring D) directly interacts with the nucleotide at position + 6 of the mRNA, which belongs to the A-site codon (20). To determine if SAR’s interactions with the ribosome are different between the model (*T. thermophilus*) and clinically relevant (*C. acnes*) organisms, we determined the structure of SAR in complex with the 70S ribosome of *C. acnes*, mRNA, and P-site tRNA at 2.8 Å resolution using cryogenic electron microscopy (cryo-EM). We characterize here unique interactions of SAR with the clinically relevant ribosome and describe structural features of the *C. acnes* ribosome with emphasis on its differences from those of *Mycobacterium smegmatis (M. smegmatis)*, which is of the same phylum (Actinobacteria) as *C. acnes*, and the model Gram-negative bacteria *E. coli* and *T. thermophilus* (13,26). Our data are important for advancing understanding of antibiotic resistance and development of narrow-spectrum antibacterial drugs, which is an urgent need for contemporary antibiotic stewardship.

## MATERIALS AND METHODS

See also Supplementary Materials and Methods.

### Antibiotics used for biochemical and structural studies

SAR was kindly provided by Almirall, LLC. TET, DOX, MIN were purchased from Millipore Sigma, USA (Cat. # T7660, D9891, M9511, respectively).

### Cell growth and ribosome purification

*Cutibacterium acnes* cells were grown in anaerobic conditions using nitrogen and CO_2_ generators (BD GasPack EZ with indicator, cat. # 260001). *C. acnes* cells (ATCC® 11827^TM^) were reactivated and plated according to ATCC instruction on blood agar contact plates (REMEL™, cat. # R111007). Cells from one plate were transferred to 6 liters flask filled with two liters of Brain Heart Infusion Broth (OXOID, cat. # CM1135) and were grown for 40 h at 37°C in shaker at 100 rpm. Identity of cells was confirmed by 16S Sanger Sequencing (CD Genomics, USA).

Ribosomes were purified from 5 g of frozen cells. Cells were suspended in 50 ml of buffer B: 20 mM HEPES–KOH, pH 7.5, 200 mM KCl, 20 mM MgAc, 1 mM DTT, 1 mg/mL lysozyme, 1 tablet of cOmplete™ Protease Inhibitor Cocktail (Roche) and 100 U of DNaseI (RNase free). Cells were lysed using microfluidizer at 15 000 psi for 3 cycles and then centrifuged at 18 000 rpm, 4°C for 30 min in Type 45 Ti rotor (Beckman Coulter). Supernatant was layered onto 25 ml sucrose cushion (20 mM HEPES–KOH, pH 7.5, 500 mM KCl, 20 mM MgCl_2_, 1 mM DTT, 1.1 M sucrose) and centrifuged at 42 000 rpm, 4°C for 21 h in the same rotor. Ribosomal pellets were suspended in high-salt buffer (20 mM HEPES–KOH, pH 7.5, 500 mM KCl, 10 mM MgAc, 1 mM DTT) by gentle shaking for 3 h at 4°C and then layered again on the sucrose cushion as described above. Ribosomal pellet was suspended in high-salt buffer and then layered onto sucrose gradient (20 mM HEPES–KOH [pH 7.5], 60 mM KCl, 10 mM MgCl_2_, 1 mM DTT, 10–40% [w/v] sucrose) to resolve 70S ribosomes by ultracentrifugation at 20 000 rpm, 4°C for 16 h in SW 32 Ti rotor (Beckman Coulter). Fractions containing 70S particles were concentrated in a spin concentrator (100 000 MWCO [molecular weight cutoff], Amicon®) and buffer-exchanged to a ribosome buffer (20 mM HEPES–KOH, pH 7.5, 60 mM KCl, 10 mM MgCl_2_, 1 mM DTT) prior to flash-freezing in liquid nitrogen.

### Preparation of re-associated 70S ribosomes

After centrifugation through the second sucrose cushion (see above) the pellet was resuspended in dissociation buffer (20 mM Tris–HCl, pH 7.4, 60 mM NH_4_Cl, 1 mM MgCl_2_, 0.5 mM EDTA, 14 mM β-mercaptoethanol (βME)), layered onto 15–40% sucrose gradient to resolve 30S and 50S subunits (20 mM HEPES–KOH [pH 7.5], 60 mM NH_4_Cl, 1 mM MgCl_2_, 14 mM βME, 15%–40% [w/v] sucrose) by centrifugation at 28 000 rpm, 4°C for 16 h in the SW 32 Ti rotor. Fractions corresponding to 50S and 30S subunits were combined, transferred to the re-association buffer (20mM Tris–HCl, pH 7.4, 60 mM NH_4_Cl, 10 mM MgCl_2_, 0.5 mM EDTA, 14 mM βME) by concentration-dilution (repeated twice) using a spin concentrator. Ribosomes were incubated at 37°C for 1 h, and then were layered onto 10–40% sucrose gradient to resolve 70S particles and collected as described above.

### Ribosomal complex formation

Reaction was done in a total volume of 20 μl with the final concentration of 0.125 μM for the re-associated 70S *C. acnes* ribosomes, 5.0 μM for mRNA (32MF), 200 μM for SAR, and a buffer composed of 20 mM Tris–HCl, pH 7.4, 60 mM NH_4_Cl, 10 mM MgCl_2_, 0.5 mM EDTA, 10 mM βME at 37°C for 30 min. Then tRNA_i_^fMet^ was added to a final concentration of 2.5 μM and the incubation was continued for another 30 minutes.

### Cryo-EM sample preparation, data collection and processing

70S-SAR–mRNA–tRNA ribosomal complex (4 μl) was applied on a 300 mesh C-Flat 2/1 3Au gold grid (Electron Microscopy Sciences, USA, cat. # CF312-50-Au) pre-treated by glow-discharging at 25 mA for 15 s. The grid was blotted (blot force –4, blot time 2 s) at 22°C with 100% humidity and plunge-frozen in liquid ethane using FEI Vitrobot Mark IV (Thermo Fisher, USA).

Images were acquired on 300 kV FEI Titan Krios electron microscope (Thermo Fisher) equipped with a post-GIF Gatan K3 direct detector in super-resolution mode, at a nominal calibrated magnification of 81 000× with a physical pixel size of 1.068 Å. The energy filter slit width was set to 20 eV. Automated data collection was set up using SerialEM (27). A total of 10 765 movie series were collected with defocus range of 0.5–2 μm. Data were collected with a dose of 16.6 electrons per pixel per second. Images were recorded over a 2.1 s exposure with 0.06 s for each frame to give a total dose of 30.56 electrons per Å^2^ split over 35 frames.

Data processing procedures were carried out using standard pipelines in cryoSPARC v3 (Supplementary Figures S2–S4, Table S1) (27). cryoSPARC ‘Blob picker’ was used on a subset of 2000 micrographs and the selected particles were used for 2D classification to generate ideal templates. These templates were used to pick particles from the entire dataset using cryoSPARC’s template picker. 1 108 852 particles were picked and extracted. After 2D classification, 911 382 particles were carried forward to generate 3D reconstructions using cryoSPARC’s Ab Initio and Heterogenous refinement jobs. Particles belonging to 3D classes representing junk particles and unbound 50S subunit were discarded and those belonging to 70S ribosome were carried forward. 117 865 particles belonging to the 70S ribosome 3D class were further subjected to *ab initio* and heterogenous refinement to separate 70S ribosome particles with and without tRNA_i_. 70 853 particles belonging to the 3D class of 70S ribosomes with bound tRNA_i_, SAR and mRNA yielded a reconstruction with an overall resolution of 2.8 Å. These 70 853 particles were then used for subvolume masking, particle subtraction, and local refinement of the 30S head, 30S body, and the 50S subunit region to generate better quality maps of these regions of the 70S ribosome [30S head (2.9 Å), 30S body (2.8 Å) and the 50S subunit (2.6 Å)] (Supplementary Figures S3 and S4).

### Model building

Initial atomic model and restraints were generated using the high resolution structure of the *E. coli* 70S ribosome, PDB ID 7K00 (26). Then the sequences of rRNA and proteins were manually converted to those of *C. acnes* using COOT (28). Some parts of the 70S ribosome of *M. smegmatis*, PDB ID 5O61, were used later to model structural elements, which are distinct from the *E. coli* ribosome but similar to the *C. acnes* one (13). The structure of SAR from our previous publication, PDB ID 6XQE, was used originally to fit the corresponding EM density (20). The EM density maps allowed us to model and refine the structure of essentially the entire *C. acnes* 70S ribosome (Supplementary Table S2). Some ribosomal proteins uL10, uL11 and uS2 at the periphery of the 70S were resolved at lower local resolution; their homology models were fitted as rigid bodies prior to refinement. The final model of the *C. acnes* 70S ribosome in complex with mRNA, tRNA and SAR was generated by multiple rounds of model building in COOT (28), followed by refinement in PHENIX (29). All figures showing atomic models were generated using PyMol software (https://pymol.org/2/).

### In-vitro translation and luciferase inhibition assays

These assays were used to measure *in vitro* ribosome activity via production of firefly luciferase in the PURExpress® Δ Ribosome Cell-free Protein Synthesis System (New England Biolabs, USA, cat. # E3313S). Cell-free extract was prepared according to manufacturer instructions. Purified 70S ribosomes from *C. acnes* or *E. coli* were added to a final concentration of 0.8 μM. Then 1 μl of an antibiotic (or peptide) was added (various concentrations) to 6.3 μl of the mixture, and after incubation for 10 min at room temperature, the reaction was supplemented with the lucRNA2caa10 mRNA (5 ng), SuperaseIN™ RNase inhibitor (2U, Ambion, cat. # AM2694) and water to make the final volume of 10 μl. The reaction proceeded for 4 hours at 37°C in the shaker and then was transferred to a 96 well white plate. The luminescent signal was detected in 1–2 min after addition of 50 μl of the Luciferase Assay Reagent (Promega, USA, cat. # E1500) using multimode plate reader TriStar LB941 (Berthold technologies LTD, UK) and analyzed using Prism 9 (Version 9.3.1) software (GraphPad Software, LLC). All experiments were done in triplicate. Translation efficiency was calculated as normalized end-point values of luminescence with measurement interval time of 0.2 s.

## RESULTS

### Overview of *C. acnes* ribosome

The overall resolution of the EM map of the 70S complex is 2.8 Å. Using local refinement on the 50S subunit and the head and body domains of the 30S subunit, we generated better quality maps of these regions at resolution 2.9 Å for the 30S head, 2.8 Å for the 30S body, and 2.6 Å for the 50S subunit (Supplementary Figures S2–S5). However, some flexible regions on the ribosome periphery were still resolved at lower local resolution and were not modeled (Supplementary Table S2). We will use the new nomenclature for ribosomal proteins (30) and *C. acnes* sequence numbering for rRNAs and proteins; corresponding *E. coli* numbering will be provided in parentheses. Helices of the small ribosomal subunit 16S rRNA are marked by ‘h’, whereas large subunit 23S rRNA helices are marked by ‘H’. Overall, the 70S ribosome structure from *C. acnes* contains 20 out of 22 ribosomal proteins of the 30S subunit, 29 out of 33 ribosomal proteins of the 50S subunit, whole 5S, 99% of 16S, and 94% of 23S rRNAs (Figure 1B, Supplementary Table S2). The EM density suggests that ribosomal proteins uS14, bL28, bL31, bL33, and bL36 are in their zinc-bound form, similar to their counterparts in the structure of *M. smegmatis* 70S ribosome (13). Like *M. smegmatis*, *C. acnes* belongs to Actinomycetia and has genes encoding isoforms for these proteins with and without Zn^2+^ binding site. It is not surprising then that structural differences between the rRNAs of *C. acnes* and the reference ribosome from *E. coli* are like the differences between *M. smegmatis* ribosome and *E. coli* ribosome (13). Therefore, in our description of the structure of the *C. acnes* ribosome, we focus predominantly on the *C. acnes*-specific differences of functionally important ribosomal proteins. Two novel ribosomal proteins, bS22 and bL37, which were recently discovered in *M. smegmatis* and are absent in *E. coli* ribosome, were present in our structure (13). We demonstrated a potential role of these proteins outside of ribosomal function. Surprisingly, we observed two binding sites for sarecycline in the *C. acnes* ribosome. In addition to the CBS, we observed a second binding site (SBS) for SAR. This site is located on the 50S ribosomal subunit within the nascent peptide exit tunnel (NPET), a major ribosome functional center (Figure 1B).

### Canonical binding site of SAR in the 30S subunit of the *C. acnes* ribosome

**Figure 2.**
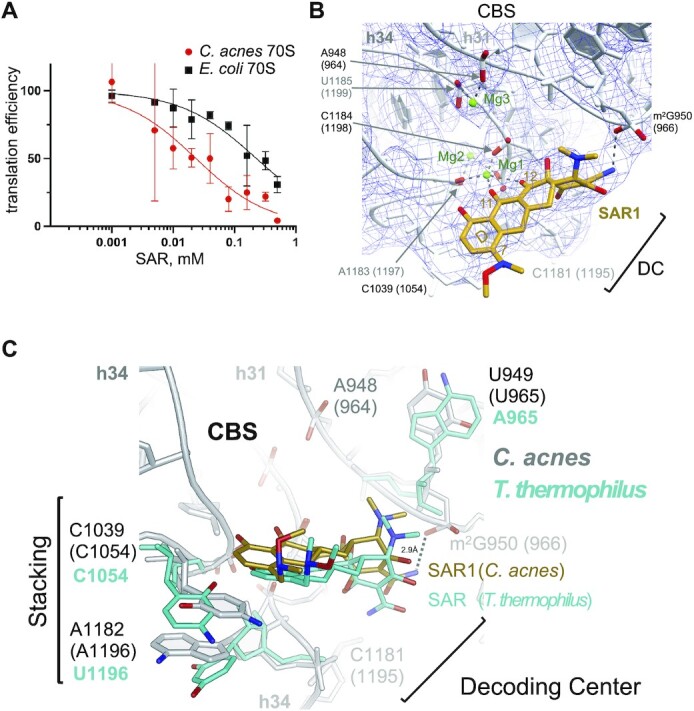
Canonical binding site of Sarecycline in the 70S ribosome from *C. acnes*. (**A**) Inhibition of protein synthesis by SAR in the cell-free system: cell- and ribosome-free translation system was supplemented with *C. acnes* 70S ribosomes (red circles) or *E. coli* 70S ribosomes (black squares). Translation efficiency was calculated as normalized end-point values of luminescence with measurement interval time of 0.2 second. Data were fitted using inhibitor vs. normalized response (variable slope) analysis model (Prism 9 software). IC50 is 0.026 ± 0.013 mM for *C. acnes* and 0.26 ± 0.10 mM for *E. coli* (mean ± standard deviation). (**B**) Canonical binding site (CBS) of SAR1 in the 30S subunit. SAR’s carbon atoms are colored gold, nitrogens are blue, oxygens are red and magnesium ions are green. Electron density map (mesh) is shown in blue. (**C**) Superposition of SAR CBS from *C. acnes* (gray) and *T. thermophilus* (PDB ID 6XQE, cyan) 70S ribosomes.

Ribosomes from *C. acnes* were tested in an *in vitro* translation assay (Figure 2A). Translation by *C. acnes* ribosomes was 10-fold more susceptible to SAR compared to *E. coli* ribosomes, with an IC_50_ of 26 ± 13 and 260 ± 100 μM, respectively. This result agrees with previously measured minimum inhibitory concentration (MIC) 0.5–16 μg/ml (1.0–32 μM) for SAR in *C. acnes*, indicating that protein synthesis machinery is the main target for SAR (16). For cryo-EM studies, considering the higher sensitivity of *C. acnes* to SAR, we lowered SAR concentration to 200 μM compared to 500 μM previously used for the crystallographic *T. thermophilus* 70S ribosomal complex. Importantly, only one SAR binding site was observed in *T. thermophilus* at 500 μM of SAR (20). The SAR binding site in *C. acnes* 30S ribosomal subunit is similar to that in the *T. thermophilus* ribosome. SAR binds in the CBS, inhibiting the decoding center (Figure 2B, Supplementary Figure S6A). SAR interacts with 16S rRNA and the overall mode of this interaction is similar to other tetracycline derivatives because their naphthacene cores share identical hydrophilic/polar edges, which face the 16S rRNA (20–23). The complex is stabilized by intermolecular hydrogen bonds between backbone phosphate oxygen atoms of C1039, A1183, and G1184 (C1054, A1197, and G1198) and oxygen atoms at C11 and C12 of SAR via a Mg^2+^ ion (Figure 2B), a feature conserved for all tetracycline/ribosome interactions. Surprisingly, the nitrogen atom of carboxamide group in ring A of SAR forms a direct hydrogen bond with the backbone phosphate oxygen atom of m^2^G950 (m^2^G966) of h31 (Figure 2B). In the crystal structure of *T. thermophilus* 70S-SAR complex this carboxamide group is rotated 180 degrees, similar to other tetracyclines, but different from SAR in *C. acnes*. Therefore, in *T. thermophilus* the interaction proceeds through the oxygen atom and is mediated by a Mg^2+^ ion, whereas the nitrogen atom is in hydrogen bonding distance with C1195 (*T. thermophilus* numbering) (20). No interaction with C1181 (C1195) was observed in our structure (Figure 2C).

Ring D of SAR stacks on nucleobase C1039 (C1054), which in turn stacks on A1182 (A1196), creating a three-layered stacking interaction. These interactions stabilize binding of the D-ring side of SAR in the decoding center (Figure 2C). They also may promote SAR’s greater antibacterial potency toward *C. acnes*, because in our previous structure of *T. thermophilus* 70S-SAR complex these stacking interactions are much less pronounced, as they are less pronounced in other structures of ribosomes with tetracyclines bound (Figure 2C) (20,22,23). However, third-generation tetracyclines like TIG and eravacycline (ERA) formed stacking interactions similar to SAR in *C. acnes* ribosome when bound to ribosomes from *E. coli*, *T. thermophilus* and *A. baumannii* (20,23–25). In the *C. acnes* structure, the SAR C7 moiety is oriented toward nucleobase + 6 of the mRNA, which may allow sequence-specific interactions with the mRNA similar to those in *T. thermophilus* 70S-SAR complex (Supplementary Figure S7) (20).

### Second binding site of SAR in the 50S subunit of the *C. acnes* ribosome

**Figure 3.**
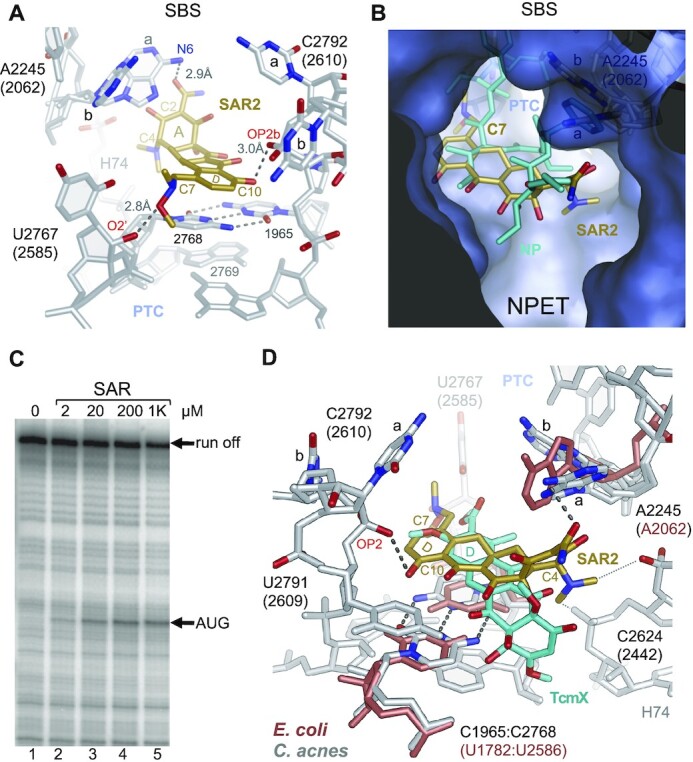
Second Binding Site of Sarecycline within the 70S ribosome from *C. acnes*. (**A**) SAR2 (gold) interacts with the 23S rRNA (gray) in the second binding site (SBS) in the 50S subunit. (**B**) View inside the nascent peptide exit tunnel (NPET). Superposition with the *E. coli* ribosome (PDB ID 7B5K) showing that SAR2 will clash with the nascent peptide (NP). (**C**) The toe-printing assay in a cell- and ribosome-free translation system supplemented with *C. acnes* ribosomes. Reverse transcriptase extends cDNA to the position on the mRNA where antibiotic stopped the translating ribosome, producing a toe-print. As expected, we detected a prominent toe-print corresponding to the ribosome stalled at the initiation AUG codon. In comparison, the *E. coli* ribosomes toe-print showed ribosomes stopped downstream of AUG, indicating that some ribosomes escaped inhibition during initiation, but were stopped later (see Supplementary Figure S9B and also Figure 6 in Batool et. al., 2020). With *C. acnes* ribosomes this pattern is rather absent (compare lane 1 with 2–5), suggesting that two molecules of SAR may act cooperatively, inhibiting decoding center, PTC and/or NPET, which would decrease the probability of transition of initiation complex to elongation. The full gel is shown in Supplementary Figure S9A. (**D**) SBS overlaps with the TetracenomycinX (TcmX) binding site. View of the SBS after superposition of *C. acnes* and *E. coli* (PDB ID 6Y69, brown) 70S ribosomal complexes. TcmX is shown in cyan.

During 50S subunit EM map analysis, we discovered electron density within the NPET that is consistent with the SAR chemical structure (Supplementary Figure S6B). The NPET is ∼100 Å long and between 10 and 20 Å wide. It starts at the PTC, where it is narrow, then passes through the body of the large ribosomal subunit and ends at an opening on the solvent side of the particle (Figure 1B). The second molecule of sarecycline (SAR2) binds in the proximal region of the exit tunnel, opposite the macrolide antibiotics binding site, near the PTC with its ring D close to the PTC and its ring A extended down the NPET towards the exit (Figure 3A, Supplementary Figure S8A). In this position SAR2 interferes with the growing peptide chain, as seen when our structure is superposed with an *E. coli* 70S ribosome complex with the P-site-bound tRNA-nascent chain (Figure 3B) (31). The oxygen of the carboxamide group attached to C2 in ring A of SAR2 makes a hydrogen bond with the N6 atom of A2245 (A2062) of the 23S rRNA. Interestingly, the local EM density map suggests two possible orientations for A2245 (A2062), conformations a and b. In conformation b, it will not clash with the nascent peptide nor interact with SAR2 (Figure 3A, B). These two conformations of A2245 (A2062) were observed in other bacteria during crystallographic and cryo-EM studies of macrolide antibiotics (32,33). Genetic, biochemical, and structural analyses revealed this universally conserved nucleotide (A2062) in the PTC loop is critical for drug-dependent ribosome stalling. In *E. coli* A2062 may act as a tunnel sensor, where its conformation is sensitive to the presence of nascent peptide sequences in the exit tunnel; it communicates with the PTC via neighboring nucleotides, making it unable to catalyze formation of the next peptide bond when a macrolide and a nascent peptide with specific amino acid sequence are present in the NPET (34–36). Conformations of (A2062) observed in the presence of macrolides and their derivatives are similar to those in our structure, indicating SAR2 may affect the activity of the PTC the same way macrolides do (Supplementary Figure S8B). The dimethyl-amino group at the C4 position of SAR2, which is common to all tetracyclines, forms van der Waals contacts with the backbone atoms of helix 74 (H74) of the 23S rRNA, additionally stabilizing binding of SAR2 (Figure 3A, D).

Ring D of SAR2 stacks on C2768 (U2586), which forms a non-Watson-Crick base pair with C1965 (U1782). To be formed, this noncanonical base pairing requires protonation of cytosine at N3, generating a positive charge that may additionally stabilize binding of an acidic SAR2 molecule (37). The hydroxyl oxygen at the C10 position in ring D forms hydrogen bond with the phosphate oxygen (OP2) of C2792b (C2610) (Figure 3A). Again, we observed two orientations for C2792 (C2610), where C2792a protrudes in the NPET, overlapping with the macrolide binding site, and C2792b (U2610) is the conformation commonly observed in bacterial ribosome structures (Supplementary Figure S8B). U2610 in *E. coli* is involved in binding of macrolides; it was proposed to be a sensor that recognizes bound macrolides and links 23S rRNA conformational changes in NPET to the PTC (34). For SAR2 it may behave the same way.

We observed direct interaction of SAR C7 moiety with 23S rRNA in the NPET. It is hydrogen bonded with the oxygen atom at the O2’ position of U2767 (U2585) (Figure 3A). Rearrangement of U2767 (U2585) upon A-site tRNA binding is required for proper binding of its aminoacyl moiety to the A site on the 50S subunit (38). In our structure, the position of U2767 (U2585) is not compatible with the correct orientation of the aminoacyl moiety of the A-site tRNA, and hence SAR2 should inhibit peptide bond formation (Supplementary Figure S8C). Therefore, our data suggest that SAR2 may work cooperatively with SAR1 in the CBS to inhibit binding of the tRNA to the A site. In the event a tRNA binds to the A site, SAR2 might inhibit peptide bond formation. However, we note there is no tRNA bound to the A site in our structure, which leaves unanswered the question of whether the SAR2 interaction with U2767 (U2585) is strong enough to keep U2767 in the observed conformation when the A site is occupied. To visualize the inhibition effect of SAR on the translating ribosome we used toe-printing analysis of the *in vitro* translated mRNA in the *E. coli* cell- and ribosome-free system supplemented with purified *C. acnes* or *E. coli* ribosomes. We see prominent toe-print at AUG (Figure 3C), and some downstream of it when *E. coli* ribosomes were used (Supplementary Figure S9B). Compared to *E. coli* ribosomes, escape from SAR translational inhibition at the start codon was less for *C. acnes* ribosomes. We speculate that two molecules of SAR may act cooperatively, contributing to inhibition of decoding center, PTC and/or NPET, decreasing the probability of transition of initiation complex to elongation.

The SBS overlaps with the recently identified binding site for the antibiotic tetracenomycin X (TcmX) in the *E. coli* ribosome (Figure 3D) (39). Both SAR and TcmX are four-ring aromatic polyketides, although of different structural families (Supplementary Figure S1). Neither SAR nor TcmX bind the 50S subunit of the *T. thermophilus* ribosome (20,39). Osterman *et al.* proposed that the noncanonical base pair U1782–U2586 in *E. coli* 23S rRNA is crucial for TcmX binding, and that the C–C base pair in the equivalent position in *T. thermophilus* confers resistance. Indeed, when a C-C base pair was introduced into the 23S rRNA of *E. coli* at that position, the MIC of *E. coli* for TcmX increased fourfold (39). In contrast to TcmX, SAR2 stacks on top of C1965-C2768 in *C. acnes* 23S rRNA. However, neither SAR nor TcmX binds *T. thermophilus* 23S rRNA, indicating that even small differences in the overall environment inside the NPET can influence drug binding. The nucleotide sequences of the 23S rRNA comprising SAR’s SBS are identical in *C. acnes* and *T. thermophilus*. However, the orientation of A2062 in *T. thermophilus* 23S rRNA is different from both a and b orientations of corresponding A2245 in *C. acnes*, and it is not compatible with the position of the carboxamide and hydroxyl groups in ring A of SAR2 (Supplementary Figure S10). Notably, the base of A2062 in *T. thermophilus* 23S rRNA is rotated towards the exit of the NPET and its orientation is stabilized by stacking interactions with histidine 69 (HIS69) in the ribosomal protein uL4 (Supplementary Figure S10). This interaction is plausibly strong enough to interfere with accommodation of ring A of SAR2 in that area of NPET. The uL4 from *C. acnes* or *E. coli* has a glycine residue instead of a histidine at that position, which allows A2245 (A2062) to move so that it can participate in the SAR2 binding. Previous studies have tested SAR activity against a panel of various Gram-positive and Gram-negative bacteria (16,40). Interestingly, all of the bacterial strains tested did not have HIS69 in their uL4 ribosomal protein and instead have a glycine in the corresponding position (Supplementary Table S3). SAR MIC measured in these studies could be influenced by a wide range of factors beyond ribosomal interactions, like cell penetration, environment, efflux pumps, ribosomal protection proteins, etc. Therefore, to elaborate if the general rule exists (aforementioned noncanonical C–C base pair and glycine instead of HIS69) for SAR2 binding in the SBS, more structures of SAR with ribosomes from different organisms are needed. Analysis of toeprinting assay data suggests that TcmX arrests translation at an early elongation step, as macrolides do (39). We propose that SAR2 may function the same way, though we did not explore if SAR2 inhibition is sequence specific.

### Ribosomal proteins of the 30S subunit with *C. acnes*-specific differences

**Figure 4.**
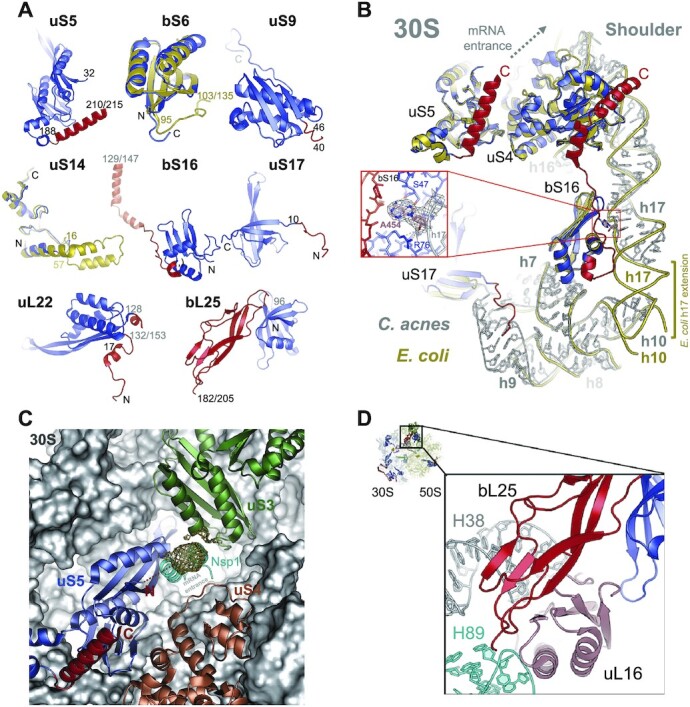
*C. acnes-*specific extensions. (**A**) Ribosomal proteins with *C. acnes*-specific features (conserved domains are in blue and *C. acnes-*specific extensions are in red). Superposed *E. coli* proteins (PDB ID 7K00) are shown in gold. N- and C- termini are marked; some amino acid sequence numbers are shown (total length of some proteins is shown after slash symbol). (**B**) *C. acnes* (gray) and *E. coli* (yellow, PDB ID 7K00) 16S rRNAs were superposed on h17. The local EM map of A454 is shown. (**C**) The C-terminal extension of uS5 has a helical structure (in red). We observe EM density (gold mesh) in the mRNA entrance, which corresponds to the N-terminal extension of uS5. The surface of the 30S subunit is shown in gray. To show position of the C-terminal domain of Nsp1 (cyan), the structure of the 40S-Nsp1 complex (PDB ID 7JQB) was superposed on the structure of *C. acnes* 30S subunit using uS5 as a reference. (**D**) *C. acnes*-specific C-terminal domain (red) of bL25 interacts with uL16 (brown) and stabilizes the binding of uL16 to the ribosome.

To reveal *C. acnes*-specific structural differences in the ribosome, we superposed our structure with the high-resolution structures of the 70S ribosomes from *M. smegmatis* and the model bacterium *E. coli* (13,26). Several *C. acnes* ribosomal proteins have pronounced differences with their *E. coli* homologues. Ribosomal proteins uS5, uS9, bS16, uS17, bL22 and bL25 have extensions on their N-terminus, C-terminus, or both, which were visualized in our structure, except for the likely flexible parts of N-termini of uS5 and uS9, and C-termini of bS16, bL22 and bL25, for which we did not observe corresponding EM density (Figures 1B, 4A, Supplementary Figure S11 and Table S2).

Compared to *E. coli*, bS16 from *C. acnes* has 65 amino acid long C-terminal extension, which binds to uS4 (Figure 4B). *C. acnes*-specific interaction between bS16 and uS4 connects the bottom and the top (the shoulder) parts of the body domain of the 30S subunit, which undergo conformational changes during translation upon binding of elongation factor Tu and tRNA (41). This connection through protein-protein interaction is absent in *E. coli* where the direct interaction between h10 and extended h17 of 16S rRNA maintains structural integrity of the body domain of the 30S subunit. Moreover, bS16 in our structure interacts with the A454 of h17 of the 16S rRNA (Figure 4B). A454 is absent in the *E. coli* 16S rRNA and would be an insertion between A452 and G453. Flipped A454 binds in the pocket formed by the conserved domain and *C. acnes*-specific loop of bS16, and anchors h17 to the protein. This way h17 is glued to h7 via bS16, providing even more rigidity to this region.

In *E. coli*, h17 is longer and directly interacts with h10, which is continuing from h7 (Figure 4B). h17 is a size-variable zone in the variable region 3 (V3) of 16S rRNA (42). Unlike all other variable regions of the 16S rRNA, size of V3 has unique bimodal distribution with peaks around 161 and 186 nucleotides (43). *C. acnes* V3, like most Actinobacteria, has a short version of V3 (Supplementary Figure S12). Though h17 is a part of a multiple helix hub, namely the 5-way junction, which is vital for the 30S ribosome assembly, its length variation did not change the base of the helix or the 5-way junction (Supplementary Figure S12) (43). It is more likely then that species-specific stabilization of V3 region by bS16 interaction with h17 plays a role in translation initiation of specific mRNAs but not in rRNA assembly, considering that h17 can function as an additional initiation site for the mRNAs with super Shine Dalgarno regions (44).

It was noted that *M. smegmatis*-specific proteins of the 30S subunit are clustered on the solvent-exposed side adjacent to the mRNA entrance (13). Among them uS5 and uS4 are involved in the binding and unwinding of secondary structures of the mRNA. Therefore, upon mRNA binding, *C. acnes*-specific extension of bS16, which interacts with uS4, may also participate in remodeling of the mRNA entrance, as was proposed for uS4 and uS5 from *M. smegmatis* and *E. coli* (13,45). Unlike in the EM map of *M. smegmatis* ribosome, we observed globular density in the mRNA entrance, which we attributed to the *C. acnes*-specific N-terminal extension of uS5 (Figure 4C). In this position, the N-terminus of uS5 would plug the mRNA channel and block the correct mRNA loading. Peculiarly, this is very similar to the function of the C-terminal domain of the nonstructural protein 1 (Nsp1) of SARS-CoV-2 (46,47). Superposition of the *C. acnes* 30S subunit and the 40S-Nsp1 structures showed that they occupy homologous sites in the mRNA binding channel (Figure 4C). It is still not clear how the plug is removed from the entrance to the channel, but uS3, uS4, and uS5 proteins surrounding the mRNA entrance are universally conserved. Thus, we hypothesize that the mechanism regulating the clearance/unplugging of the mRNA entrance, as used by *C. acnes* or hijacked by the virus in human cells, is conserved. The N-terminal *C. acnes*-specific extension of uS17 provides additional connectivity to the network of aforementioned proteins and the 16S rRNA regions. In *C. acnes*, the N terminus of uS17 interacts with the major groove of h9, unlike in the *M. smegmatis* ribosome, where it binds between h7 and h9 (Figure 1B, Supplementary Figure S13) (13).

uS9 has a 46 amino acid long N-terminal extension (23 amino acids longer than *M. smegmatis*) and is located on the opposite side of the mRNA binding channel, above the mRNA exit site (Figure 1B, Supplementary Figure S14). We observed EM density for the N-terminal part of uS9 starting from Lys40, because the 39 amino acid long terminus was disordered likely due to its flexibility (Supplementary Figure S11). Another ribosomal protein adjacent to the mRNA exit site, bS21, is involved in mRNA binding during translation initiation in *E. coli* (48). This protein is absent in some bacteria, including *M. smegmatis* (13). We observed a weak EM density in the place of its possible location in the *C. acnes* ribosome. However, local resolution of the EM map does not allow us to build a model. The shape of that density does not correspond to that of *E. coli* bS21. We also were not able to identify a bS21 counterpart in the *C. acnes* genome using NCBI database and the basic local alignment search tool (BLAST®). Therefore, this density may correspond to a 3’end of the 16S rRNA (nucleotides 1522–1537), which can be located in this area.

Two proteins of the 30S subunit, bS6 and uS14, are shorter than their *E. coli* counterparts (Figure 4A). The 40 amino acid long C-terminal extension of *E. coli* bS6 is located on the 30S solvent side. It was shown in the ribosome from *Flavobacterium johnsoniae* that the 3’-end region of the 16S rRNA, including the anti-Shine-Dalgarno sequence, binds along a groove formed by bS18 and the C-terminal region of bS6 (15). That C-terminal region is present in *E. coli* but not in the *C. acnes* bS6, which may reflect some differences between these organisms in the mechanism of translation initiation. The ribosomal protein uS14, which we observed in our structure, is 61 amino acids long and binds to a Zn^2+^ ion (Supplementary Figure S15). The *C. acnes* genome also contains another uS14 variant, this one similar to *E. coli* because it does not have a Zn^2+^-binding motif. Like its *E. coli* homologue, this variant is 101 amino acids long (Supplementary Figure S16). Most bacteria have only one variant of uS14. It was proposed that the loss of the Zn^2+^-binding motif evolved to adapt bacteria to zinc-limited environments (49). Similar to *M. tuberculosis*, *C. acnes* genome has both zinc and zinc-free variants for six ribosomal proteins (Supplementary Table S2). In *M. tuberculosis* zinc depletion induces ribosome hibernation in part through substitution of Zn^2+^-binding ribosomal proteins with their zinc-free paralogues; moreover, this process may enhance the pathogen's antibiotic tolerance and result in a pathogen subpopulation called ‘persisters’, which resist/survive antibiotic treatment (50,51). Interestingly, review and meta-analysis of available data showed that patients with acne vulgaris have significantly lower level of serum zinc compared to controls (52). Treatment of acne patients with zinc resulted in clinical improvement and was more efficient if used in combination with topical antibiotics (52). Considering that the antibiotics used in these treatments were inhibitors of ribosomal function, we hypothesize that treatment with zinc prevents *C. acnes* substitution of Zn^2+^-binding ribosomal proteins for their zinc-free paralogues, thereby precluding *C. acnes* from switching its ribosomes to a hibernation mode and from establishing a population of persister bacteria. In other words, antibiotic therapy for acne vulgaris may work better when *C. acnes* is uniformly in its Zn^2+^-bound state.

### Ribosomal proteins of the 50S subunit with *C. acnes-*specific differences

Several proteins of the large ribosomal subunit of *C. acnes* are significantly different in size compared to their counterparts from *E. coli* and even from *M. smegmatis*. uL4 has the solvent exposed C-terminal extension, which is 100 amino acids longer than in *E. coli* and 86 amino acids longer than in *M. smegmatis*. However, there is no density for it in the EM map, because it is likely disordered.

**Figure 5.**
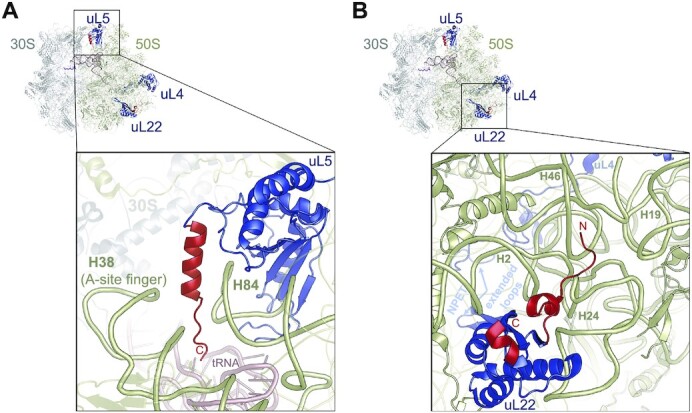
*C. acnes-*specific extensions of the large ribosomal subunit proteins. (**A**) The C-terminal region of uL5 extends between helices H38 and H84 of the 23S rRNA (khaki). P-site tRNA is colored in pink; the 30S subunit is in gray. C-terminus marked by C. (**B**) The C-terminal region of uL22 extends toward the solvent. N-terminal part binds in the area between helices H2, H24, H46 and the tip of H19 of the 23S rRNA (khaki). uL4 (on the back) is colored in light blue. Loops of uL22 and uL4 extended in the nascent peptide exit tunnel (NPET) are marked by arrows. Conserved domains of uL5 and uL22 are colored in blue and *C. acnes*-specific extensions are in red.

The 31 amino acid long C-terminal extension of uL5 is also disordered in the EM map of the 70S ribosome. However, after local refinement of the 50S subunit we traced the C-terminus of uL5 in the space between the tips of the functionally important helices 84 (H84) and 38 (A-site finger) of the 23S rRNA (Figure 5A). In *E. coli*, the A-site finger of the 23S rRNA is crucial for the stringent response factor, RelA, to bind the ribosome (53). Bacterial stringent response is an important stress-response pathway regulating bacterial virulence and antibiotic resistance. RelA binds the ribosome in response to amino acid deprivation. If uncharged tRNA (deacylated) is bound to the A site of the ribosome, RelA is activated and synthesizes (p)ppGpp, which regulates transcription of genes involved in stress response, replication, and growth (53,54). The A-site finger interaction with H84 via the C-terminal extension of uL5 may stabilize/affect the conformation of the RelA binding site and therefore regulate the *C. acnes*-specific stress response.

The solvent-exposed C-terminal extension of uL22 is visible in our structure. It is 43 amino acids long, similar to uL22 in *M. smegmatis*; however, in that structure it was disordered (Figures 1B, 4A, 5B) (13). The N-terminal extension anchors together helices H2, H24, H46 and the tip of H19 of the 23S rRNA (Figure 5B). Extended loops of uL22 and uL4 constrict the nascent peptide exit tunnel (NPET), affecting the ribosome's interaction with the nascent peptide and macrolides/ketolides antibiotics (55,56). Numerous mutations described in uL4 and in the carboxy-terminal region of uL22 result in decreased susceptibility to macrolides and ketolides (57). *C. acnes* specific extensions of uL4 and uL22 can allosterically affect conformation of the extended loops of these proteins, changing the geometry of NPET and its interaction with the nascent peptide and any bound antibiotic, providing species-specific sensitivity to drugs.

*C. acnes* bL25 protein comprises two structural domains, similar to those in *T. thermophilus* and *M. smegmatis*, whereas bL25 from the *E. coli* ribosome contains only a single domain (Figures 1B, 4A, Supplementary Figure S11) (13,58,59). bL25 stabilizes the binding of the functionally important uL16 on the ribosome (60). The additional C-terminal domain in *C. acnes* bL25 increases bL25–uL16 contact area and further stabilizes the binding of uL16 to the ribosome (Figure 4D). uL16 together with helices 89 (H89) and 91 (H91) of the 23S rRNA directly interact with orthosomycin antibiotics evernimicin and avilamycin, which inhibit accommodation of the A-site tRNA (60). Considering differences in the binding stability of uL16 to the 23S rRNA and interactions of the *C. acnes*-specific protein bL37 with H89 (see below), the orthosomycin binding site might be a target for further development of species-specific antibiotics.

### Ribosomal protein bS22

**Figure 6.**
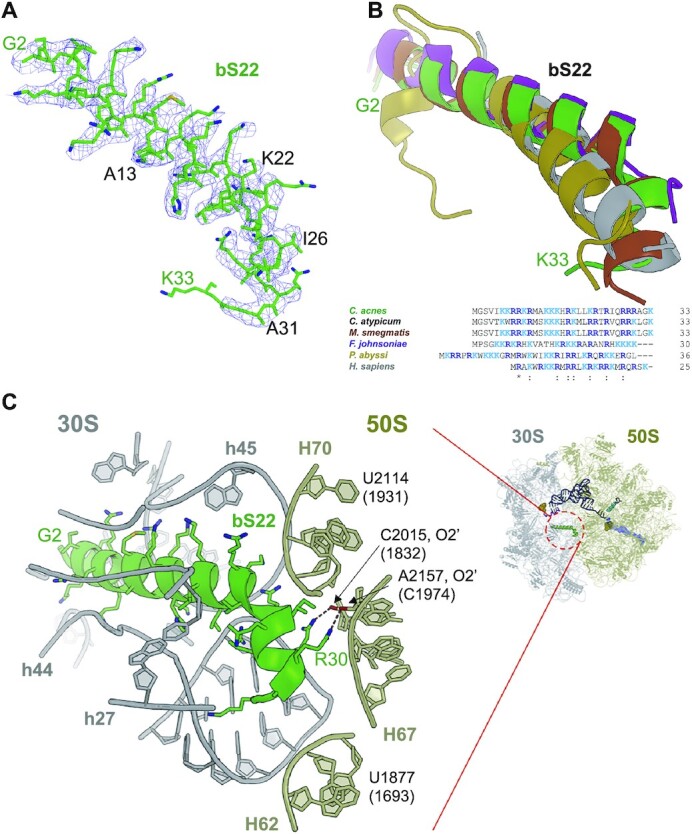
Overview of the bS22 Binding Site within the *C. acnes* 70S ribosome. (**A**) Model of the *C. acnes* bS22 (green) perfectly fits unassigned density in the EM map (blue). Residues which are different from *M. smegmatis* bS22 are marked in black. (**B**) Sequence alignment and structure superposition of bS22 and eL41 from bacteria, archaea, and human. Protein's color matches the name's color in the alignment. (**C**) Binding site of bS22 within the 16S (gray) and 23S (khaki) rRNAs.

The starting model for fitting the *C. acnes* ribosome structure was the high-resolution structure of the *E. coli* 70S ribosome (PDB ID 7K00) (26). It became evident from the unassigned, strong EM density that a helical protein is bound to the 30S subunit deep in the cavity between 16S rRNA helices h27, h44 and h45 (Figure 6A, B). This was reminiscent of the structure of the rabbit 40S ribosomal subunit where we discovered strong electron density in the same location and attributed it to the eukaryotic large ribosomal subunit protein 41 (eL41) (61). The homology search of the *C. acnes* protein sequences database with the human eL41 did not produce any hits. Therefore, we superposed available small ribosomal subunit structures to identify if that location is occupied in other ribosomes. Indeed, we found that human 55S mitochondrial ribosome (PDB ID 7NSI) and *Mycobacterium smegmatis* 70S ribosome (PDB ID 5O61) have proteins bound to this site (13,62). Amino acid sequences of both mitochondrial and *M. smegmatis* proteins, mS38 and bS22, respectively, were used to search in *C. acnes* database via NCBI protein BLAST (63). The short 33 amino acid long protein, annotated as AURKAIP1/COX24 domain-containing protein, was the best hit and we successfully modeled its amino acid sequence into the corresponding EM density (Figure 6A). Moreover, *M. smegmatis* ribosome has a second unique protein, bL37, EM density for which is also present in the map of the *C. acnes* ribosome (see below). Interestingly, bS22 was recently observed in the structure of the 70S ribosome from *Flavobacterium johnsoniae*, which represents a large, understudied group of Bacteroidetes (15). The sequence identity of *C. acnes* bS22 with that of *M. smegmatis* and *F. johnsoniae* is 85% and 37%, respectively, and they superposed well in the context of the 30S subunit (Figure 6C). Including eL41, these proteins contain 55–65% of positively charged amino acid residues and therefore are highly basic (p*I* = 12.9 for *C. acnes* bS22). These positively charged amino acid residues are involved in multiple interactions not only with the 16S rRNA, but also with helix H67 of the 23S rRNA (Figure 6B). The latter interactions form a new inter-subunit bridge, which provides additional stability to the 70S ribosome (13). The N-terminal part of bS22 interacts with h44 and h45 of the 16S rRNA at the neck of the 30S subunit, which serves as a rotation point of the head domain during translation initiation and elongation. This ensures correct interaction of the mRNA and the ribosome, including interactions in the A site (64). Therefore, it is possible that bS22 allosterically affects the inhibitory activity of SAR1 bound to CBS, which is in the A site, by influencing the way it interacts with the mRNA.

### Ribosomal protein bL37

**Figure 7.**
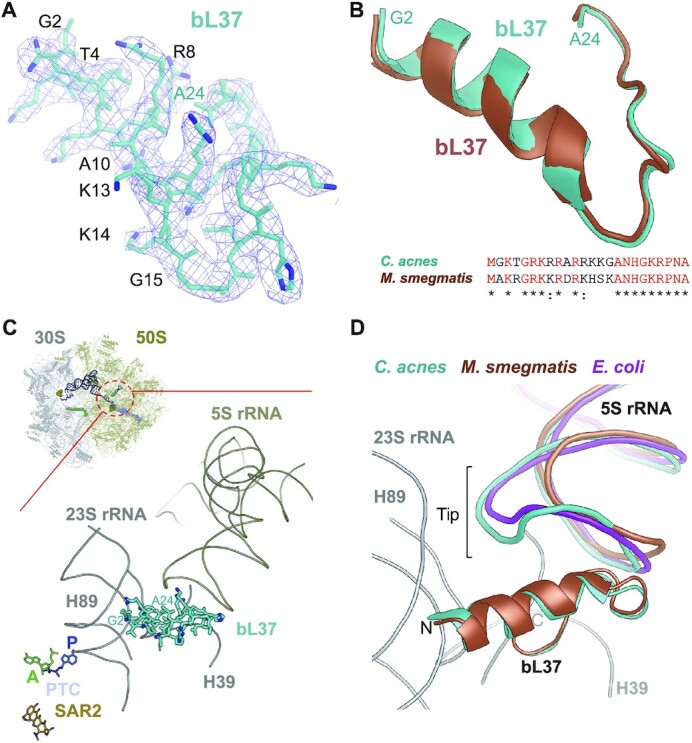
Overview of the bL37 Binding Site within the *C. acnes* 70S ribosome. (**A**) Model of the *C. acnes* bL37 (cyan) perfectly fits unassigned density in the EM map (blue). Residues which are different from *M. smegmatis* bL37 are marked in black. (**B**) Amino acid sequence alignment and structural superposition of bL37. (**C**) Binding site of bL37 within the 23S (gray) and 5S (khaki) rRNAs. (**D**) Same as **C**, with bL37 and 5S rRNAs from superposed structures of the *M. smegmatis* (brown) and *E. coli* (magenta) ribosomes. Proteins and 5S rRNAs colors match the name's colors on the figures.

During model building of the *C. acnes* ribosome we also discovered additional unassigned EM density in the area below the tip of the 5S rRNA (Supplementary Figure S5). The quality of the density allowed us to build the model for the protein and identify its amino acid sequence, which was annotated in the NCBI database as hypothetical protein FB41_0958 (*Cutibacterium acnes*) (Figure 7A). Similar to bS22, this ribosomal protein called bL37 is also present in the ribosomes of *M. smegmatis and M. tuberculosis*, but was not observed in the structures of other bacterial ribosomes published (13,65). *C. acnes* bL37 has the same protein length and structure as bL37 from *M. smegmatis* and shares 67% sequence identity (Figure 7B). It is located in the cavity beneath the tip of the 5S rRNA that is near the PTC and interacts with the 23S and 5S rRNAs (Figures 1B, 7C, Supplementary Figure S5). Interestingly, the tip of the *C. acnes* 5S rRNA is one nucleotide longer than that in *E. coli* and significantly larger in size compared to that in *M. smegmatis* (Figure 7D, Supplementary Figure S17). The N-terminus of bL37 interacts with the phosphate-backbone of H89 of the 23S rRNA, which constitutes a part of the PTC and stabilizes tRNAs binding in the A and P sites of the 50S subunit (Figure 7C) (66). It was suggested that bL37 contributes to the structural stability of this critical part of the ribosome and may affect the activity of oxazolidinone class of antibiotics, which bind to the 50S subunit rRNA pocket near the peptidyl-transferase loop of 23S rRNA domain V and inhibit peptidyl transfer (13). We propose that bL37 has even broader function: it connects together H39, H40, H72, H89, and the tip of the 5S rRNA. 5S rRNA links together and facilitates communication between the functional centers of the ribosome (67). Therefore, bL37 may act as a 5S rRNA extension in coordinating all of the functional centers of the ribosome. Moreover, considering that bL37 is not present in all bacteria and the interaction with the tip of the 5S rRNA is variable in size, bL37 may provide distinct, species-specific response of the ribosome to environmental signals like, for example, antibiotic binding.

### Antimicrobial activity of bS22 and bL37

Ribosomal proteins bS22 and eL41 resemble short α-helical peptides, are highly basic, and bind the small ribosomal subunit in the same location. Moreover, eL41 not only copurified with the rabbit and human 40S subunit in high salt conditions, but also co-crystallized with it in one to one molar ratio (based on intensity of the corresponding density in the electron density map) (61,68). Archaeal homologue aL41 also copurified with the small ribosomal subunit and its binding site resembles that of the bacterial one (69). Therefore, we strongly believe that these proteins belong to the same group of small ribosomal subunit proteins and should be named as universal S22 (uS22).

**Figure 8.**
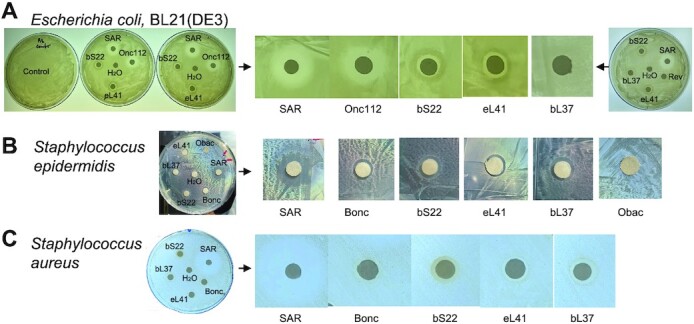
Antimicrobial activity of bS22 and bL37. Disk diffusion assay was performed in replicas (shown in A) for: (**A**) *E. coli* cells. (**B**) *S. epidermidis* cells. (**C**) *S. aureus* cells. Onc112 and Bonc are antimicrobial peptides (positive control); Obac is the inactive derivative of Onc112 (negative control). Central disk has water (solvent control).

**Figure 9.**
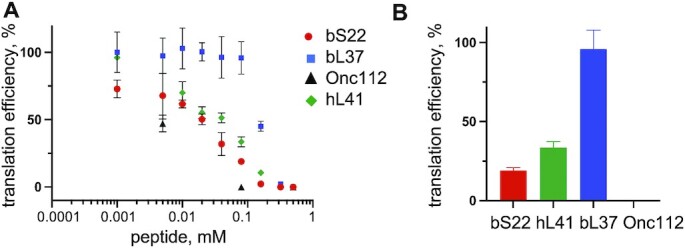
Antimicrobial activity of bS22 and bL37. (**A**) Inhibition of protein synthesis by peptides in the *E. coli* cell-free system. (**B**) same as A, at 0.08 mM concentration of the peptide. The firefly luciferase inhibition assay was done in triplicate. Translation efficiency was calculated as normalized end-point values of luminescence.

Recently, activity of human eL41 outside of its ribosomal function was discovered. Human eL41 functioned as a cell-penetrating peptide, interacting with ATF4 and targeting it to the cytoplasmic proteasome for degradation (70–72). We consider bS22 as a structure-functional homologue of eL41 and therefore investigated if free bS22 also has functions outside the ribosome. Because bS22 poses strong positive charge and has large percentage of hydrophobic amino acid residues, which are common characteristics of α-helical antimicrobial peptides (73), we tested bS22 from *C. acnes* for antimicrobial activity against *E. coli, Staphylococcus epidermidis*, and the skin pathogen *Staphylococcus aureus* using the Kirby-Bauer assay (disk diffusion assay) (Figure 8). bS22 inhibits cell growth of all these bacteria, though for *E. coli* cells inhibition is not as strong as the one caused by proline-rich antimicrobial peptide Onc112 (Figure 8A) (74). Interestingly, bL37 exhibits growth inhibition activity against Gram-positive *S. epidermidis and S. aureus*, but not towards Gram-negative *E. coli* (Figure 8). Next, we examined whether these ribosomal proteins inhibit protein synthesis in a cell-free system. Indeed, *C. acnes* bS22 and human eL41 inhibit protein synthesis in the *E. coli* cell-free system, but bL37 does not (Figure 9A). At a concentration of 80 μM, bS22 and eL41 inhibit protein synthesis at levels of 80% and 65%, respectively, whereas bL37 demonstrated very low protein synthesis inhibition (Figure 9B). These results demonstrate that *C. acnes* ribosomal proteins bS22 and bL37 may have their own functions outside ribosomal protein synthesis, in particular playing a role in maintaining homeostasis of the skin/pilosebaceous unit microbiome through their antimicrobial activity. Moreover, bS22 can function in this pathway as a broad-spectrum inhibitor, while bL37 appears to have specificity toward Gram-positive bacteria.

## DISCUSSION

Highly conserved both in sequence and three-dimensional structure, ribosomes of most eubacteria bind the majority of antibiotics the same way. The result is many antibiotics inhibit the growth of a wide range of bacteria. Such broad-spectrum activity can cause unintended negative effects on the gut and skin microbiota, which harm the patient's health and quality of life (2,3). Narrow-spectrum antibiotics can reduce side effects of this sort, and reduce the rate at which drug resistant strains of bacteria emerge (5), thereby promoting the clinical concept of antibiotic stewardship. To identify unique interactions of the narrow-spectrum antibiotic sarecycline with its clinical target, the *C. acnes* ribosome, we determined the structure of the 70S ribosome of *C. acnes* in complex with mRNA, P-site tRNA and SAR. The structure revealed distinguishing features of the *C. acnes* 70S ribosome and an unexpected second SAR binding site in the active center of the 50S subunit, not previously observed for antibiotics in the tetracycline class.

Most of the 30S ribosomal proteins with *C. acnes*-specific extensions (uS5, bS16, and uS17) bound near the mRNA entry site of the 30S subunit. In addition, the N-terminus of uS5 likely blocks mRNA entry into the mRNA channel. We propose these extensions are required for regulation of translation initiation and mRNA translocation. In addition, *C. acnes*-specific C-terminal extension of uL5 may be involved in regulation of adaptation to changes in environmental conditions, including stress and antibiotic susceptibility, because it interacts with the A-site finger of the 23S rRNA—the binding site for the stringent response factor RelA. Further characterization of all steps of *C. acnes* protein synthesis is required to reveal specificity of its structural framework and its mechanism of regulation. This is important for the development of new targeted/narrow-spectrum activity antibiotics to treat human diseases associated with *C. acnes*, including acne vulgaris, but also other less well-known complications like implant-associated infections, chronic blepharitis, and endophthalmitis (75).

We discovered Actinobacterium-specific ribosomal proteins bS22 and bL37 in our structure (13). bS22 contacts the neck of the head domain of the 30S subunit and may therefore affect 30S head's mobility, which is important for the proper positioning and movement of mRNA through the ribosome. bL37 is close to the PTC and NPET and interacts with the 5S rRNA, participating in the structural integrity of this crucial part of the ribosome. Therefore, we propose bS22 and bL37 support *C. acnes*-specific mechanisms of protein synthesis and may influence *C. acnes*-specific binding of sarecycline. Moreover, we demonstrated potential activity of these proteins outside of ribosomal function. Both bS22 and bL37 inhibit growth of Gram-positive *S. epidermidis* and the pathogen *S. aureus*; however, only bS22 inhibits growth of Gram-negative *E. coli*. The skin surface is home to a diverse population of microorganisms, including bacteria, fungi, and viruses. Between them are beneficial microorganisms, which prevent the invasion of pathogens, support our immune system, and metabolize some natural products (2). *C. acnes* is a lipophilic bacterium, which together with other *Propionibacterium* species dominates in sebaceous sites. There it coexists with the commensal clades of *Staphylococcus*, such as *S. epidermidis* and *S. hominis*, and Corynebacterium (2,76). We demonstrated that bS22 and bL37 may penetrate the bacterial cell wall and inhibit protein synthesis, which leads us to suggest that they can work as bacteriocins, maintaining homeostasis of the skin microbiome and protecting the *C. acnes* niche.

We showed that SAR inhibits protein synthesis uniquely in its clinical target, the Gram-positive anaerobic bacterium *C. acnes*, by binding two functionally important ribosome centers/active sites. This distinguishes the mechanism of action of SAR in *C. acnes* from the Gram-negative bacterium *T. Thermophilus*, where SAR has only one ribosomal binding site. SAR’s binding to the A-site of the 30S subunit (or CBS) allows it to inhibit translation initiation, and its binding to NPET on the 50S subunit (or SBS) may enable it to inhibit the early stages of peptide elongation. The SAR C7 methoxy(methyl)amino methyl group helps anchor SAR to NPET on the side opposite to where macrolide antibiotics bind. Thus, the overall mechanism by which SAR binding to the SBS contributes to protein synthesis inhibition may be similar to macrolides, which narrow the diameter of NPET and obstruct the elongation of the nascent peptide (36). We previously demonstrated that the SAR C7 group generates substantial steric clash with Tet(M), helping SAR overcome excision by ribosomal protection proteins (20). Here, we demonstrate that the low rate of *C. acnes* resistance to SAR (10^−10^ at 4–8 MIC) may also stem from the dual binding of SAR to the *C. acnes* ribosome (16). Ribosomal mutations are less effective against SAR because they are needed in both the 30S and 50S subunits simultaneously to prevent SAR function in the two active sites. The structural basis for translational inhibition of the *C. acnes* 70S ribosome is a major advance for understanding and treating one of the top 8 most prevalent human diseases, and this molecular knowledge can foster development of future narrow-spectrum, low resistance, and pathogen-focused antimicrobials.

## DATA AVAILABILITY

Accession numbers for the cryo-EM maps of the *C. acnes* 70S ribosomal complex, local refined 50S subunit with tRNA and SAR2, local refined 30S subunit head domain with SAR, local refined 30S subunit body domain are: EMD- 26959, EMD-27009, EMD-27011, EMD-27028, respectively. Corresponding accession numbers (PDB ID) for the refined coordinates of the atomic structures are: 8CRX (70S ribosomal complex), 8CVM (50S-tRNA-SAR2), 8CVO (30S head), 8CWO (30S body). We have deposited the raw EM data in the EMPIAR database with the annotation ID EMPIAR-11362.

## Supplementary Material

gkad103_Supplemental_File
