# Supplementary material for: Sarecycline inhibits protein translation in Cutibacterium acnes 70S ribosome using a two-site mechanism
Source: Nucleic Acids Res. 2023 Mar 2;51(6):2915–30. doi: 10.1093/nar/gkad103 (PMC10085706; doi:10.1093/nar/gkad103)
Supplement: gkad103_Supplemental_File [file gkad103_supplemental_file.pdf]

Supplementary Information for

Sarecycline inhibits protein translation in *Cutibacterium acnes* 70S ribosome using a two-site mechanism

Ivan B. Lomakin[1,†*], Swapnil C. Devarkar[2,†], Shivali Patel[2], Ayman Grada[4], and Christopher G. Bunick[1,2,3*].

1. Department of Dermatology, Yale University School of Medicine, New Haven, Connecticut 06520, USA.
2. Department of Molecular Biophysics and Biochemistry, Yale University, New Haven, Connecticut 06511, USA.
3. Program in Translational Biomedicine, Yale University School of Medicine, New Haven, Connecticut 06520, USA.
4. Department of Dermatology, Case Western Reserve University School of Medicine, Cleveland, OH 44106, USA

* To whom correspondence should be addressed. Tel: +1 (203) 577-1050; Fax: +1 (203) 756-4697; Email: christopher.bunick@yale.edu. Correspondence may also be addressed to Ivan B. Lomakin. Tel: +1 (203) 577-1050; Fax: +1 (203) 756-4697; Email: ivan.lomakin@yale.edu.

† The authors wish it to be known that, in their opinion, the first two authors should be regarded as Joint First Authors.

This file includes:

Materials and Methods
Figures S1 to S17
Tables S1, S2, S3

MATERIALS AND METHODS

DNA, Plasmids, primers and RNAs.

Luciferase T7 control DNA (Promega, cat. # L4821) was used for PCR (CloneAmp HIFI PCR Premix, cat. # 639298) to generate the DNA template used for transcription of the mRNA (lucRNA2caa10) and for the sequence reference in the toe-printing assay.

Primers for PCR and sequencing (Integrated DNA Technologies, USA) were:

5'-primer:

atcTAATACGACTCACTATAGCAACAACAACAACAACAACAACAACAACAAGAAGGAGATATACCATGGAAGACGCCAAAAACATAAAG

3'-primer:

CAAAAAACCCCTCAAGACCCGTTTAGAGGCCCCAAGGGGTTATCCTAGGATCCTCATTACACGGCGATCTTGCCGCCCTTCTTGGCC

Same primer was used for toe-printing and sequencing:

pr4pcrLuc: 5' - GCCCATATCGTTTCATAGCTTCTGCC

The mRNA (lucRNA2caa10) transcription reaction and RNA purification was done using MEGAscript™ T7 Transcription Kit (Ambion, cat. # AMB13345) and MEGAclear Kit (Ambion, cat. # AM1908), respectively, following protocols provided by manufacturer.

mRNA (32MF) 5'- CAAGGAGGUAAAA**AUG**UUUUAA was synthesized by Dharmacon™ (Thermo Scientific, USA).

Cell growth.

Staphylococcus epidermidis (ATCC® 12228™) and *S. aureus* (gift from Dr. Palm, Yale University) were grown according to ATCC recommendations: ATCC Medium 3, aerobic conditions at 37°C. *Cutibacterium acnes* cells were grown in anaerobic conditions using nitrogen and CO2 generators (BD GasPack EZ with indicator, cat. # 260001). *C. acnes* cells (ATCC® 11827™) were reactivated and plated according to ATCC instruction on the blood agar contact plates (REMEL™, cat. # R111007). Cells from one plate were transferred to 6 liters flask filled with two liters of Brain Heart Infusion Broth (OXOID, cat. # CM1135) and were grown for 40 hours at 37°C in shaker at 100 rpm. Cells were collected by centrifugation at 5000 rpm at 4°C for 25 min. using Fiberlite™ F6-6 x 1000y fixed-angle rotor. Pelleted

cells were washed in the ice-cold buffer A (20mM Tris HCl, pH 7.4, 100 mM NH4Cl, 10 mM MgCl2, 0.5 mM EDTA, 10 mM β-mercaptoethanol), re-centrifugated and frozen in liquid nitrogen. Identity of cells was confirmed by 16S Sanger Sequencing (CD Genomics, USA).

Toe-Printing assay.

Ribosomal complexes formation for toe-printing reactions were carried out in 5 µL aliquots. 2 µl of Solution A and 0.6 µl of the factors mix (PURExpress® ∆ Ribosome Cell-free Protein Synthesis System, New England Biolabs, USA, cat. # E3313S) were mixed with 0.1 µl of SuperaseIN™ RNase inhibitor (2U, Ambion, cat. # AM2694), 0.5 µl of antibiotic (various concentrations) and 1 µl of the *C. acnes* or *E. coli* 70S ribosomes (8 µM). After incubation for 10 minutes at 37°C, 0.5 µl of mRNA (lucRNA2caa10, 1 µM) was added and incubation continued for 20 minutes at 37°C. 1 µl of ^{32}P end labeled primer (pr4pcrLuc, about 2.5 µM) was added to the reaction, incubated for 2 minutes at 37°C.To start cDNA synthesis 4 µl of RT-mix was added (0.5 µl of dNTP (10 mM each), 2 µl of AMV (5x) buffer, 1 µl of AMV RT (Promega, USA, cat. # M5101) and 0.5 µl of H_2O) to the reaction and incubated for 30 minutes at 37°C. Thereafter 40 µl of H_2O was added to the reaction and nucleic acids were deproteinized with 50 µl of phenol-chloroform-isoamyl alcohol mixture (Millipore-Sigma, USA, Cat. # 77617, pH 8.0). To degrade RNA, 1 µl of KOH (3 M) was added to each tube, incubated for 5 minutes at 95°C, then cooled at 4˚C for 2 min. Thereafter, 1µl of HCl (3M) was added to neutralize the reaction. For ladder generation, RT was carried out using Thermo Sequenase cycling kit (Thermo Cat. 785001KT) and 300ng of PCR template according to manufacturer's instructions. To precipitate all reactions, 1/10 volume of Na-Acetate (3 M, pH 5.2), 0.5 µl of glycogen (Invitrogen) were added to each tube, mixed and combined with 3-4 volumes of ethanol. Precipitation was done overnight at -20°C. cDNA pellets were dissolved in 10 µl of loading buffer (82 % (v/v) deionized formamide, 0.16 % (w/v) xylene cyanol (XC), 0.16 % (w/v) bromophenol blue (BB), 10 mM EDTA, pH 8.0) and then 3 µl were loaded on a denaturing 6% Polyacrylamide gel. The gel was dried, exposed to phosphor screens overnight and scanned using a Typhoon RGB Biomolecular imager (Cytiva).

Protein sequences alignment.

Protein sequences alignment was done using online CLUSTAL O(1.2.4) software at https://www.ebi.ac.uk/Tools/msa/clustalo/.

FIGURES AND TABLES

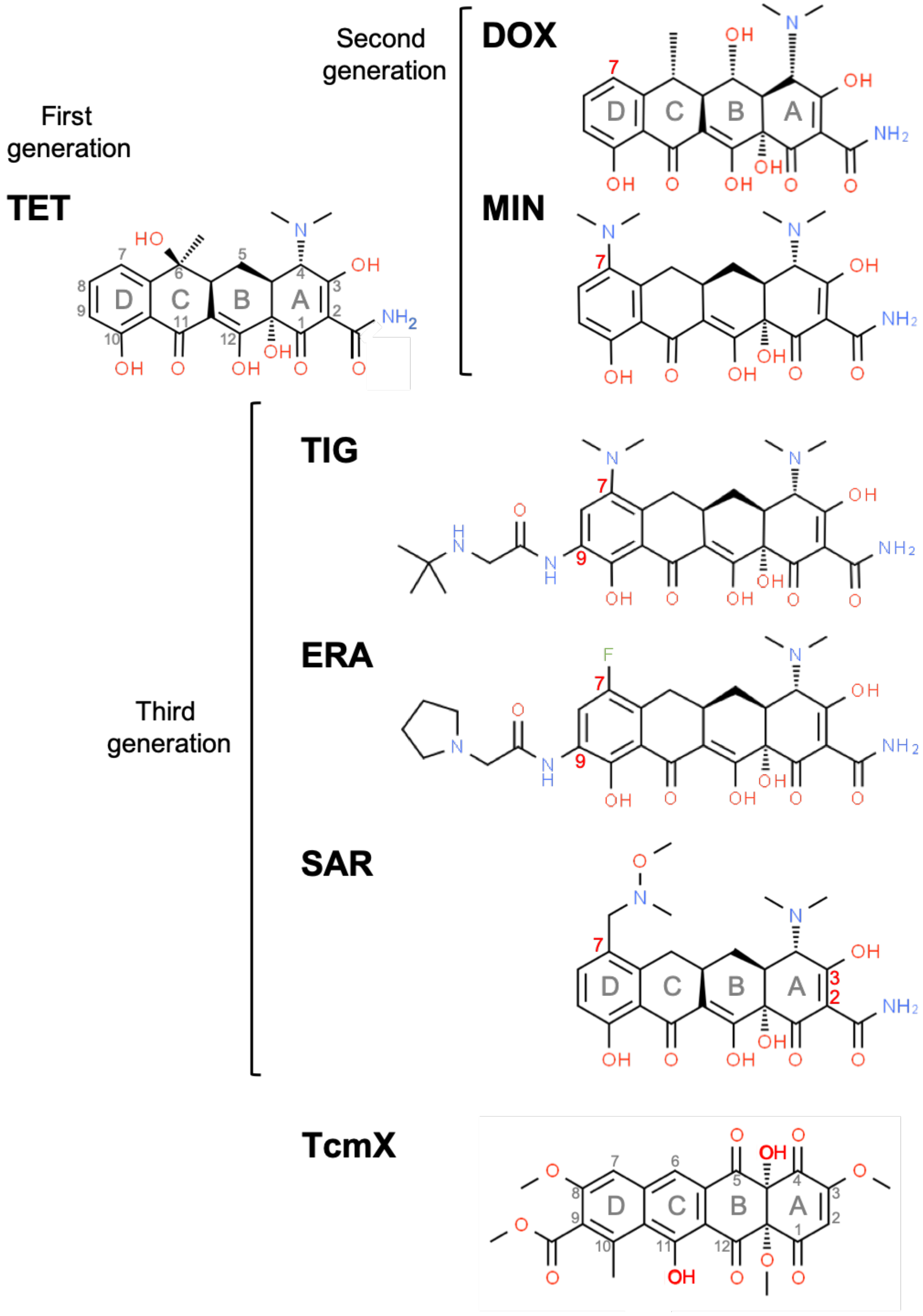

Figure S1. Chemical structures of tetracyclines and tetracenomycin X (TcmX). TET - tetracycline, DOX - doxycycline, MIN - minocycline, TIG - tigecycline, ERA - eravacycline, SAR - sarecycline.

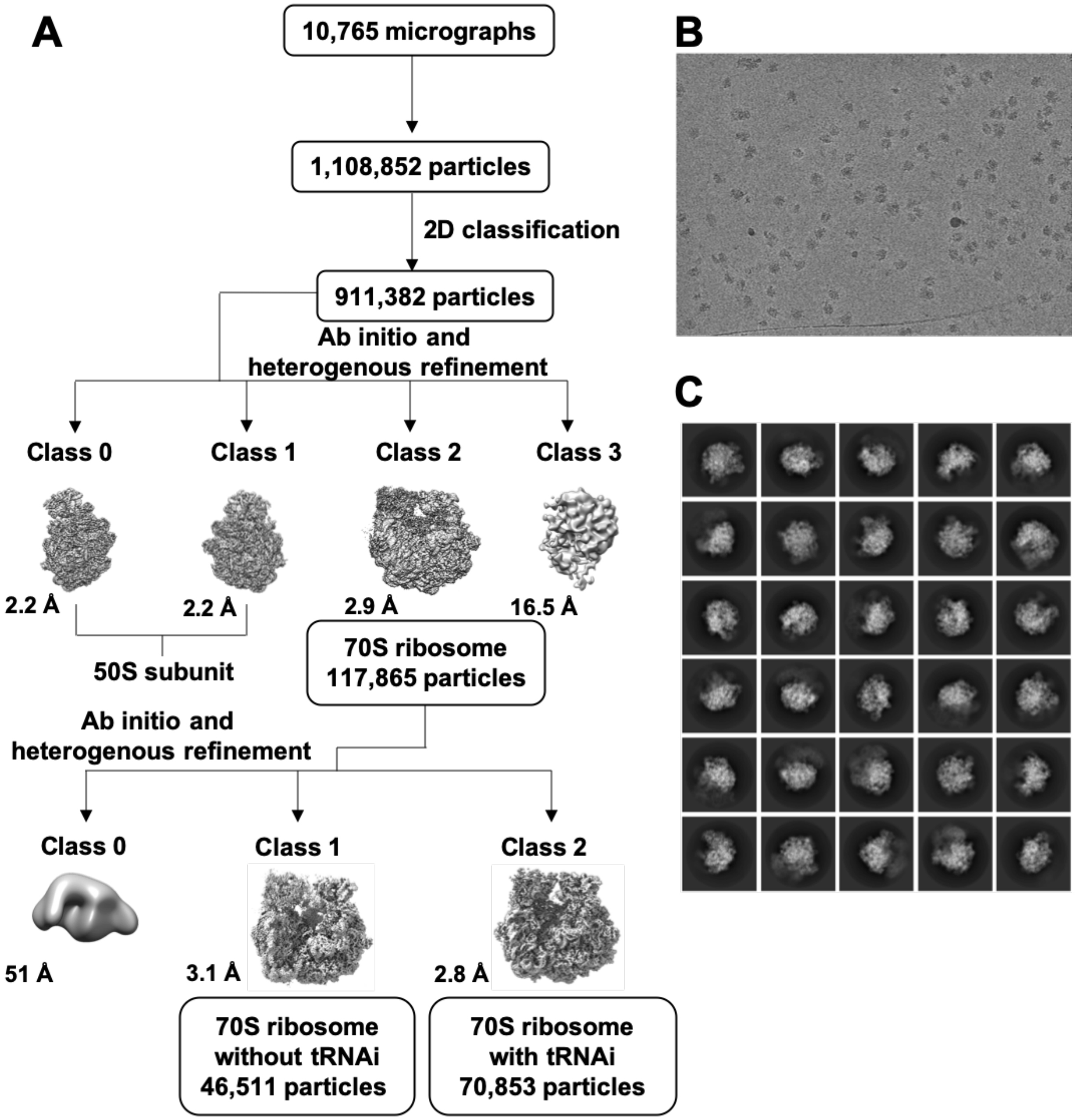

Figure S2. Cryo-EM Data Processing Workflow. (**A**) cryoSPARC 'Blob picker' was used on a subset of 2000 micrographs and the selected particles were used for 2D classification to generate ideal templates. These templates were used to pick particles from the entire dataset using cryoSPARC's template picker. 1,108,852 particles were picked and extracted. After 2D classification, 911,382 particles were carried forward to generate 3D reconstructions using cryoSPARC's Ab Initio and Heterogenous refinement jobs. Particles belonging to 3D classes representing junk particles and unbound 50S subunit were discarded and those belonging to 70S ribosome were carried forward. 117,865 particles belonging to the 70S ribosome 3D class were further subjected to ab initio and heterogenous refinement to separate 70S ribosome particles with and without tRNAi. 70,853 particles belonging to the 3D class of 70S ribosomes with bound tRNAi, mRNA and SAR yielded a reconstruction with an overall resolution of 2.8 Å that was used for model building. (**B-C**) A representative motion-corrected micrograph (**B**) and representative 2D classes (**C**) from the dataset are shown.

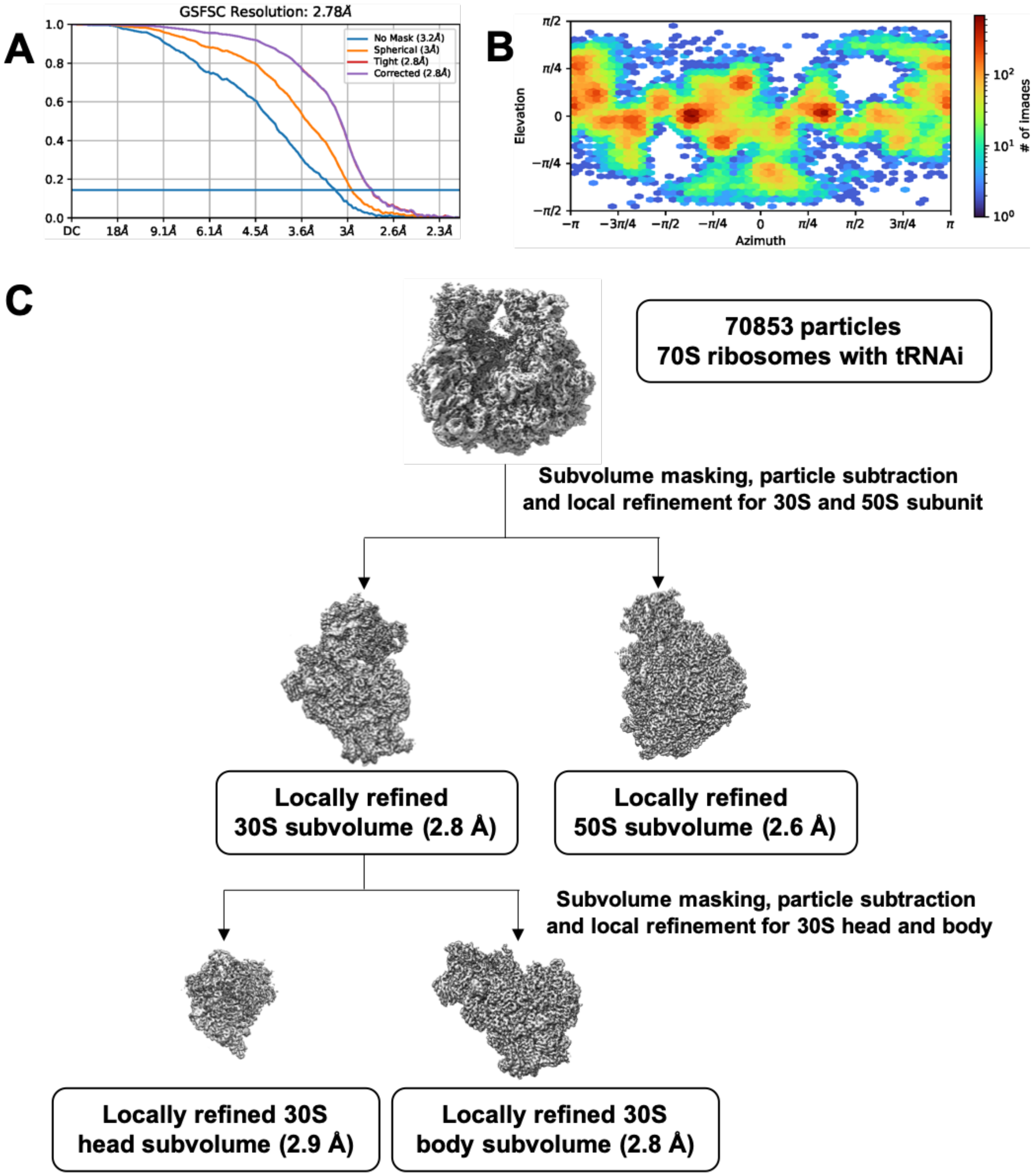

Figure S3. Focused Refinement of tRNAi bound 70S ribosome reconstruction. (**A**) Gold-standard Fourier shell correlation (GS-FSC) curve for the tRNAi bound 70S ribosome reconstruction. (**B**) Directional distribution map for the 70,853 particles used in the tRNAi bound 70S ribosome reconstruction showing the angular distribution of particle projections. (**C**) 70,853 particles belonging to the 70S ribosome class with bound tRNAi, mRNA and SAR were further processed in cryoSPARC for focused refinement. Initially the densities corresponding to the 30S and 50S subunit were masked separately and subtracted to generate two subsets of particles containing signal from either the 30S or 50S subunit. These signal subtracted particle sets were used to locally refine the 30S and 50S subvolumes, yielding EM density maps with significantly better quality for the dynamic regions of the 70S ribosome. The 50S signal subtracted 30S particles were further used for particle subtraction and local refinement of the 30S head and 30S body subvolumes.

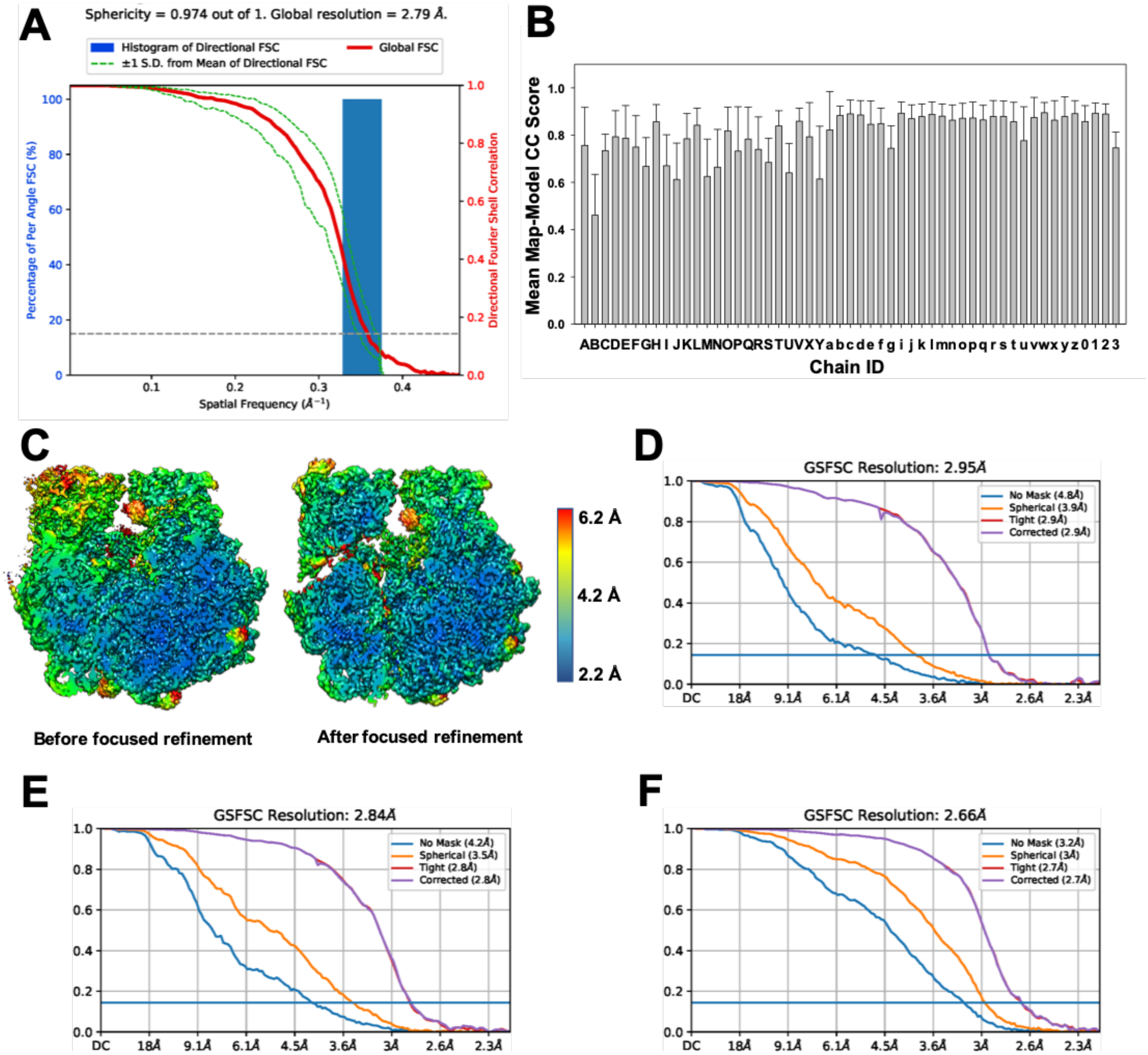

Figure S4. Cryo-EM Map and Model Validation Statistics. (**A**) 3D-FSC plot for the tRNAi bound 70S ribosome reconstruction showing the directional resolution anisotropy. An overall sphericity of 0.97 shows that the reported global resolution of 2.8 Å is uniform in three dimensions and the sample does not show any preferred orientations. (**B**) Map-model correlation coefficients per residue per chain were calculated using PHENIX. The mean correlation coefficient (Mean CC) for all the residues in every chain in the model and the associated standard deviation is presented in a bar chart form. (**C**) The local resolution heat maps for the 70S ribosome reconstructions refined globally (left panel) and using local refinement for 30S head, body and 50S subunit regions (right panel) is shown. The represented views are cross-sections of the representative EM densities. The local resolution varies from 2.2 Å for the core regions to 5-6 Å for the peripheral dynamic regions of rRNAs and ribosomal protein segments. (**D-F**) Three regions of the 70S ribosome reconstruction were locally refined as outlined in Supplementary Figure S3. Gold-standard Fourier shell correlation (GS-FSC) curves for the locally refined 30S head subvolume (**D**), 30S body subvolume (**E**) and 50S subvolume (**F**) are shown.

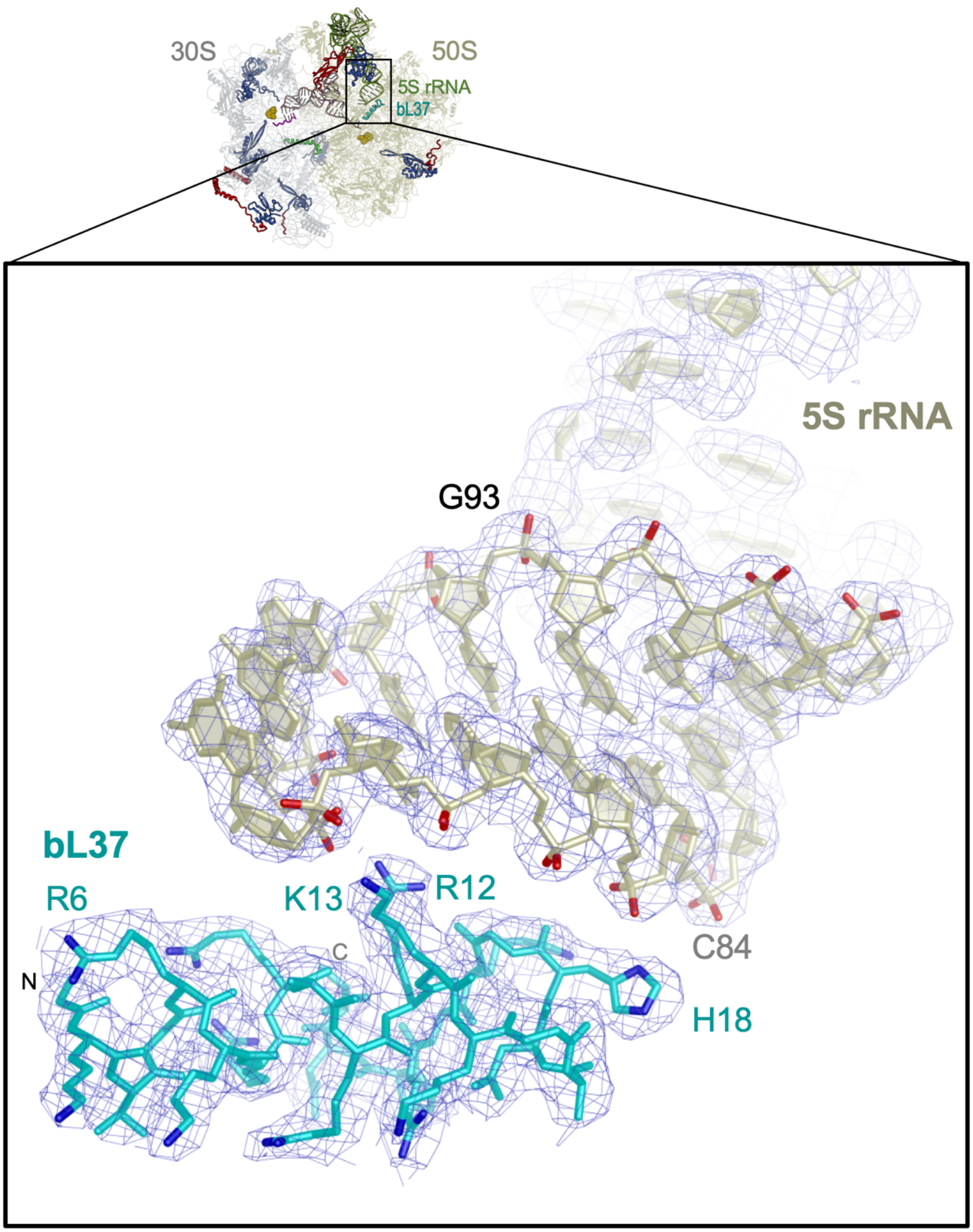

Figure S5. Interaction of bL37 with the 5S rRNA. Positively charged amino acid residues of bL37 (cyan) interact with the backbone of the 5S rRNA (khaki). Electron density map is shown in blue. Nitrogen's atoms are colored in blue and oxygens are in red.

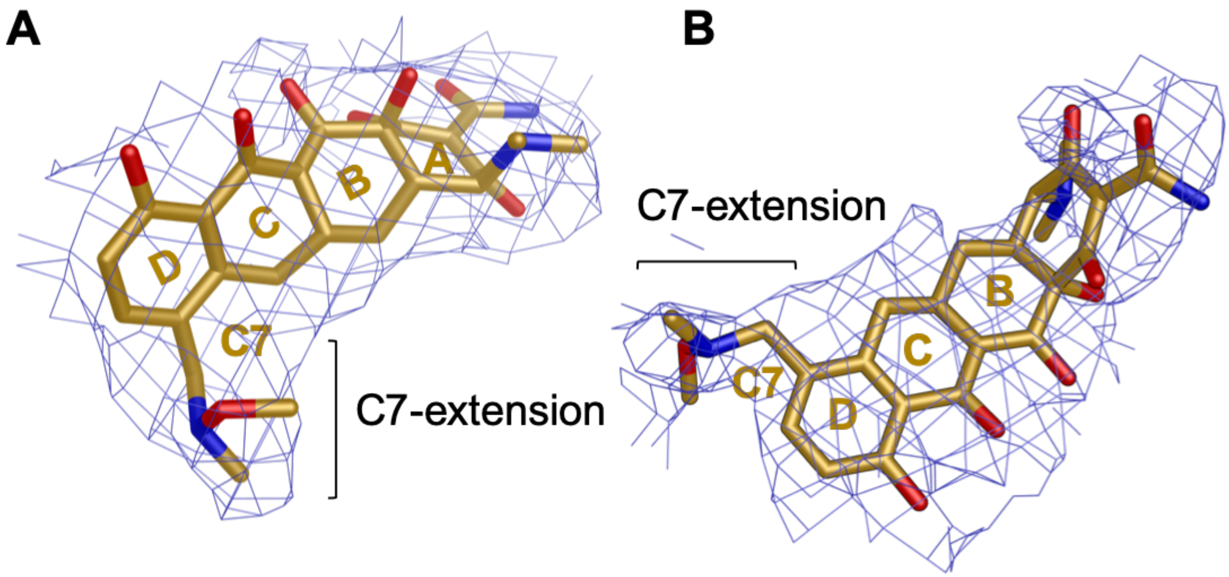

Figure S6. Structure of sarecycline bound to the 30S and 50S ribosomal subunits. (**A**) SAR1 bound in the canonical binding site (CBS) on the 30S subunit. (**B**) SAR2 bound in the second binding site in the 50S subunit. EM maps of SAR in complex with the *C. acnes* 70S ribosome are shown in blue. Carbon atoms are colored gold, nitrogens are blue and oxygens are red.

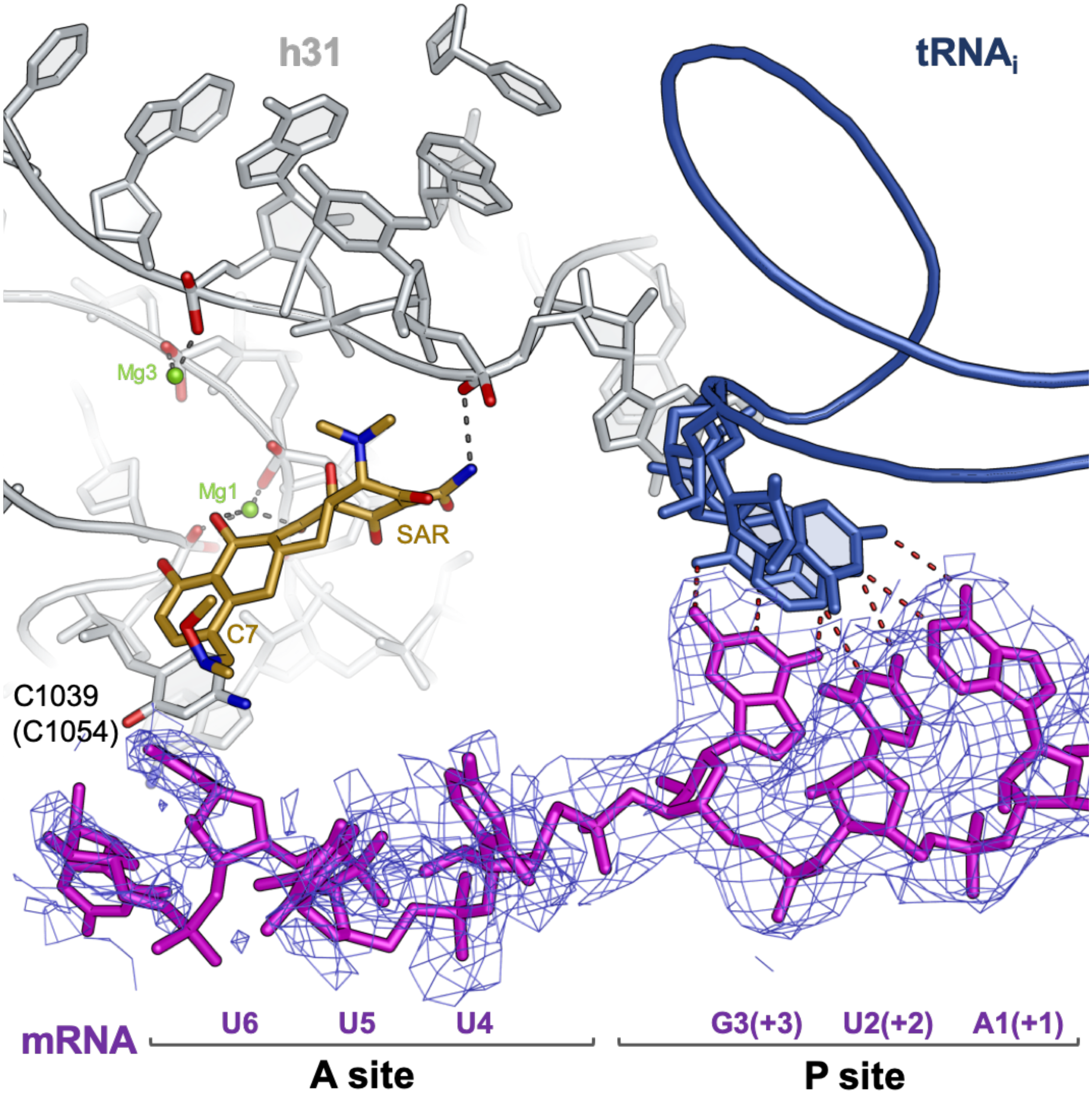

Figure S7. View from the mRNA binding channel of Sarecycline bound in CBS. The C7 moiety of SAR in the *C. acnes* 70S ribosome structure is oriented toward nucleobase of mRNA, that is likely in position +6 (the third nucleotide of the A-site codon), similar to what we observed in the *T. thermophilus* 70S-SAR complex. The quality of the EM density for mRNA diminishes after position +3. This is likely due to the high mobility of the head domain of the 30S subunit and because the mRNA is located on the border separating the head and the body domains.

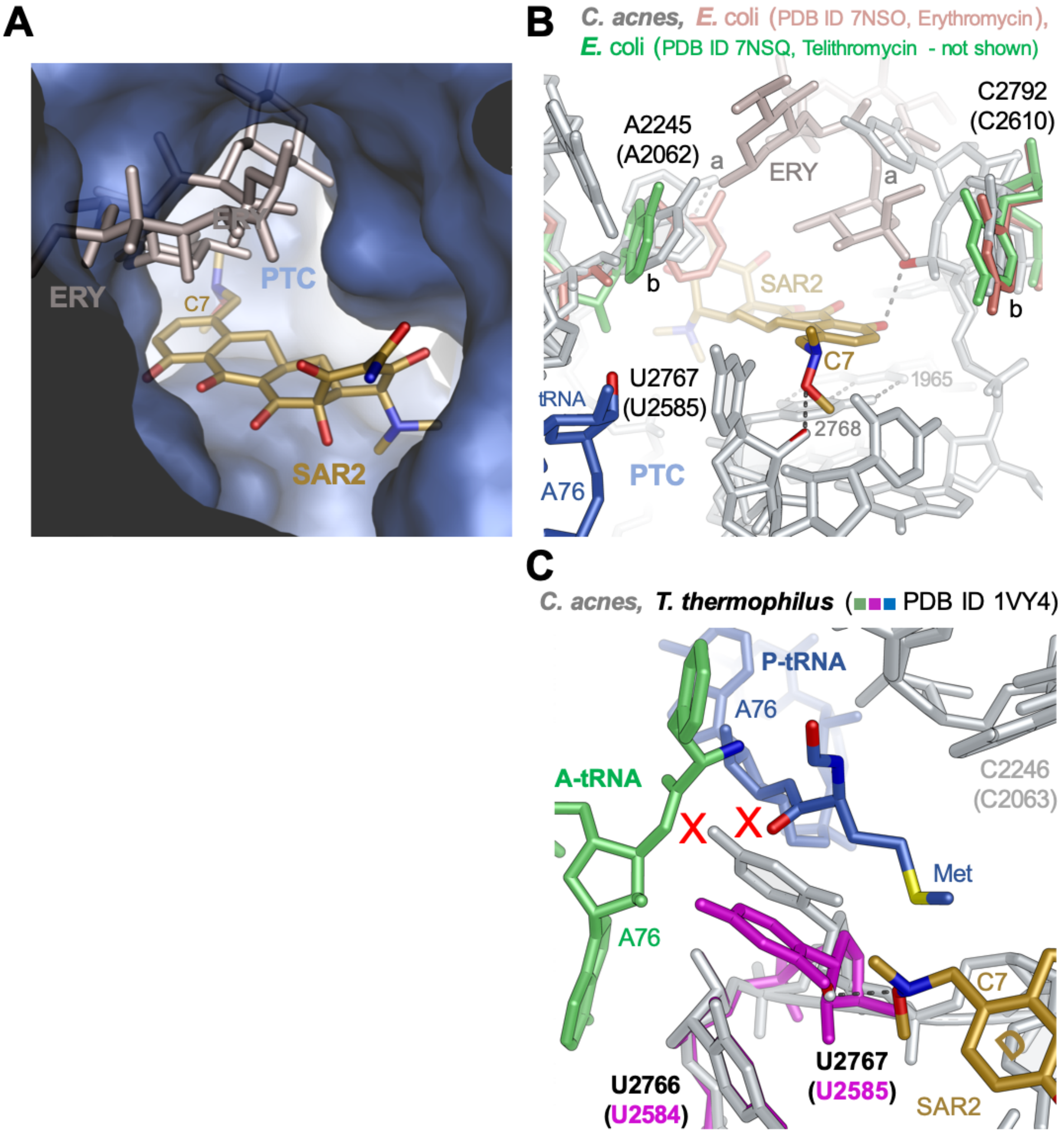

Figure S8. Second Binding Site of Sarecycline. (**A**) SAR2 (gold) binds in the exit tunnel, opposite to the macrolide antibiotics binding site (PDB ID 7NSO, ERY in light brown), in the vicinity of the PTC (**B**) Superposition of *C. acnes* (gray) and *E. coli* (PDB ID 7NSO, 7NSQ; brown and green, respectively) 70S ribosomal complexes with macrolides. (**C**) The close-up view of the ring D of SAR2 and PTC after superposition with the structure of the *T. thermophilus* 70S ribosomal complex (magenta), containing A-site (green) and P-site (blue) tRNAs in the pre-attack state of peptide bond formation. Possible clashes in the *C. acnes* 70S-SAR structure are marked by red X.

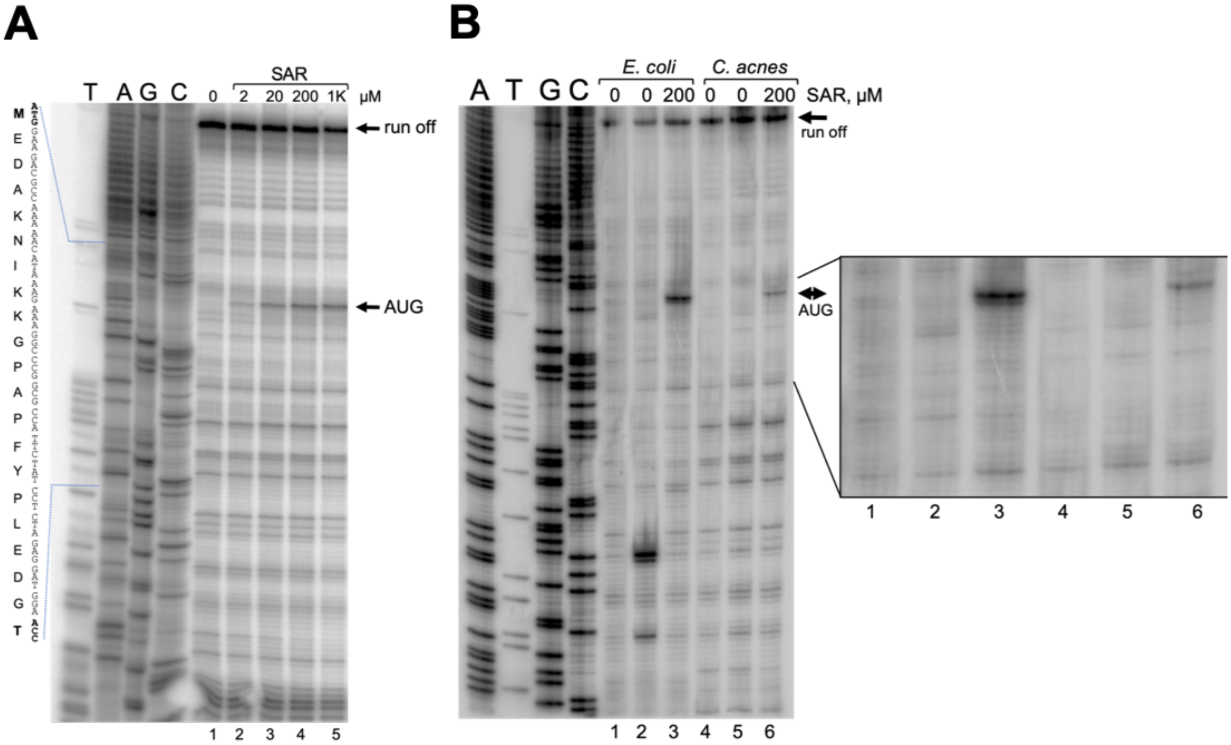

Figure S9. Ribosomes stalling by Sarecycline on lucRNA2caa10. (**A**) **The toe-printing assay in a cell- and ribosome-free translation system supplemented with *C. acnes* ribosomes.** Reverse transcriptase extends cDNA to the position on the mRNA where antibiotic stopped the translating ribosome, producing a toe-print. As expected, we detected a prominent toe-print corresponding to the ribosome stalled at the initiation AUG codon. (**B**) **The toe-printing assay in a cell- and ribosome-free translation system supplemented with *E. coli* (lanes 1-3) or *C. acnes* ribosomes (lanes 4-6).** The *E. coli* ribosomes toe-print (lane 3) showed that some ribosomes stopped downstream of AUG, indicating that some ribosomes escaped inhibition during initiation, but were stopped later (see also Fig.6 in Batool et. al., 2020). With *C. acnes* ribosomes (lane 6) this pattern is rather absent (compare lanes 3 and 6). This suggests that the two molecules of SAR may act cooperatively, contributing to inhibition of decoding center, PTC and/or NPET, decreasing the probability of transition of initiation complex to elongation. The reverse transcriptase stops at the nucleotide +16 relative to the first codon located in the P site of the ribosome. The translation reactions were supplemented with 50 µM of borrelidin, an inhibitor of threonyl-tRNA-synthetase, in lanes 2 and 5 (panel B).

A

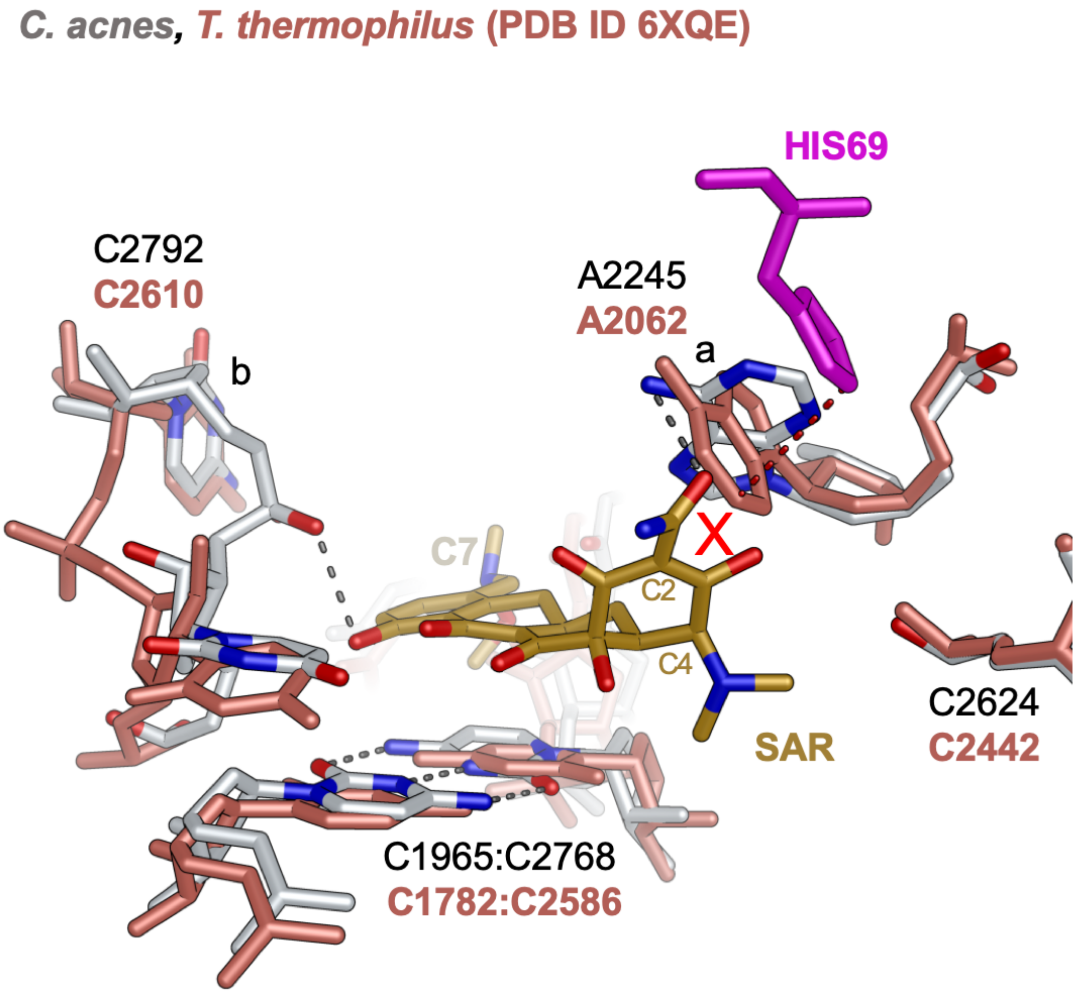

B

```
                           ▼
C. acnes         50 GTHATKTRGQVSGGGKKPWRQKGTGRARQGSTRAPQWVGGG 90
E. coli          42 GTRAQKTRAEVTGSGKKPWRQKGTGRARSGSIKSPIWRSGG 82
M. smegmatis     48 GTHSTKTRGEVSGGGKKPYRQKGTGRARQGSTRAPQFTGGG 88
T. thermophilus  47 GTASTKTRGEVAYSGRKIWPQKHTGRARHGDIGAPIFVGGG 87
                    **   ***  *   * *   ** ***** *    *   **
                                          ▲
                                        HIS69
```

Figure S10. (**A**) **Structural differences within SBS area between *C. acnes* and *T. thermophilus* 50S ribosomal subunits.** Close-up view of the SAR2's C2 moiety interaction with the 23S rRNA. Structure of *C. acnes* 50S subunit (gray) was superimposed with that of the *T. thermophilus* (PDB ID 6XQE, brown). Histidine 69 of the ribosomal protein uL4 of the *T. thermophilus* ribosome is shown in magenta. Potential steric clashes are marked by red X. (**B**) **Ribosomal protein uL4 amino acid sequences alignment within the vicinity of HIS69 (*T. thermophilus*).** Protein sequences of uL4 were aligned using Clustal O (1.2.4) software (https://www.ebi.ac.uk/Tools/msa/clustalo/). Accession numbers: *C. acnes* - NR_075172.1, *E. coli* - NR_075284.1, *M. smegmatis* - PDB ID 5O61, *T. thermophilus* - PDB ID 6XQE.

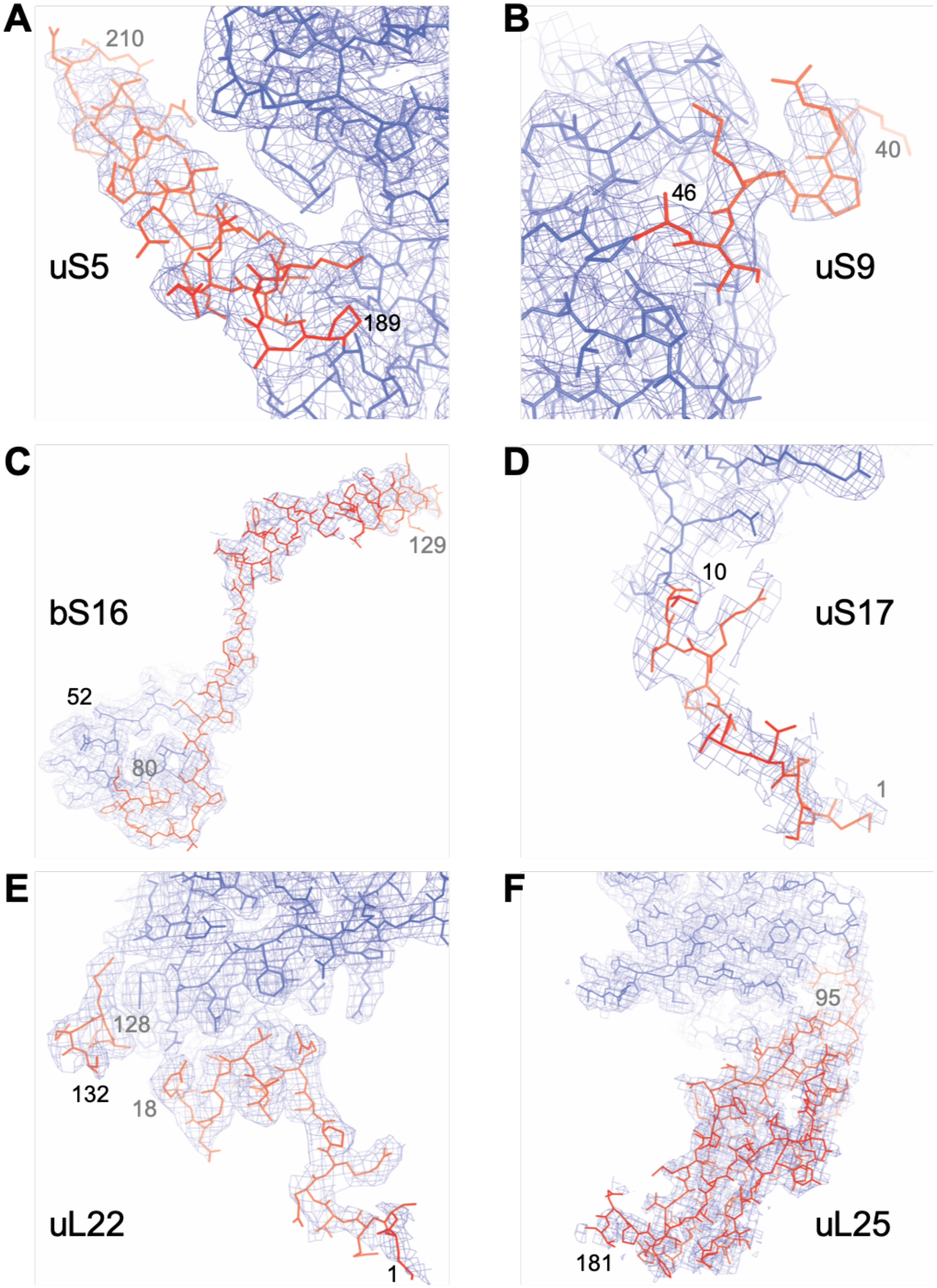

Figure S11. *C. acnes*-specific features of ribosomal proteins: (**A**) **uS5.** (**B**) **uS9.** (**C**) **bS16.** (**D**) **uS17.** (**E**) **uL22.** (**F**) **uL25.** Conserved domains are in blue and C. acnes-specific extensions are in red. Some amino acid sequence numbers are shown as reference markers. Electron density map (mesh) is shown in blue.

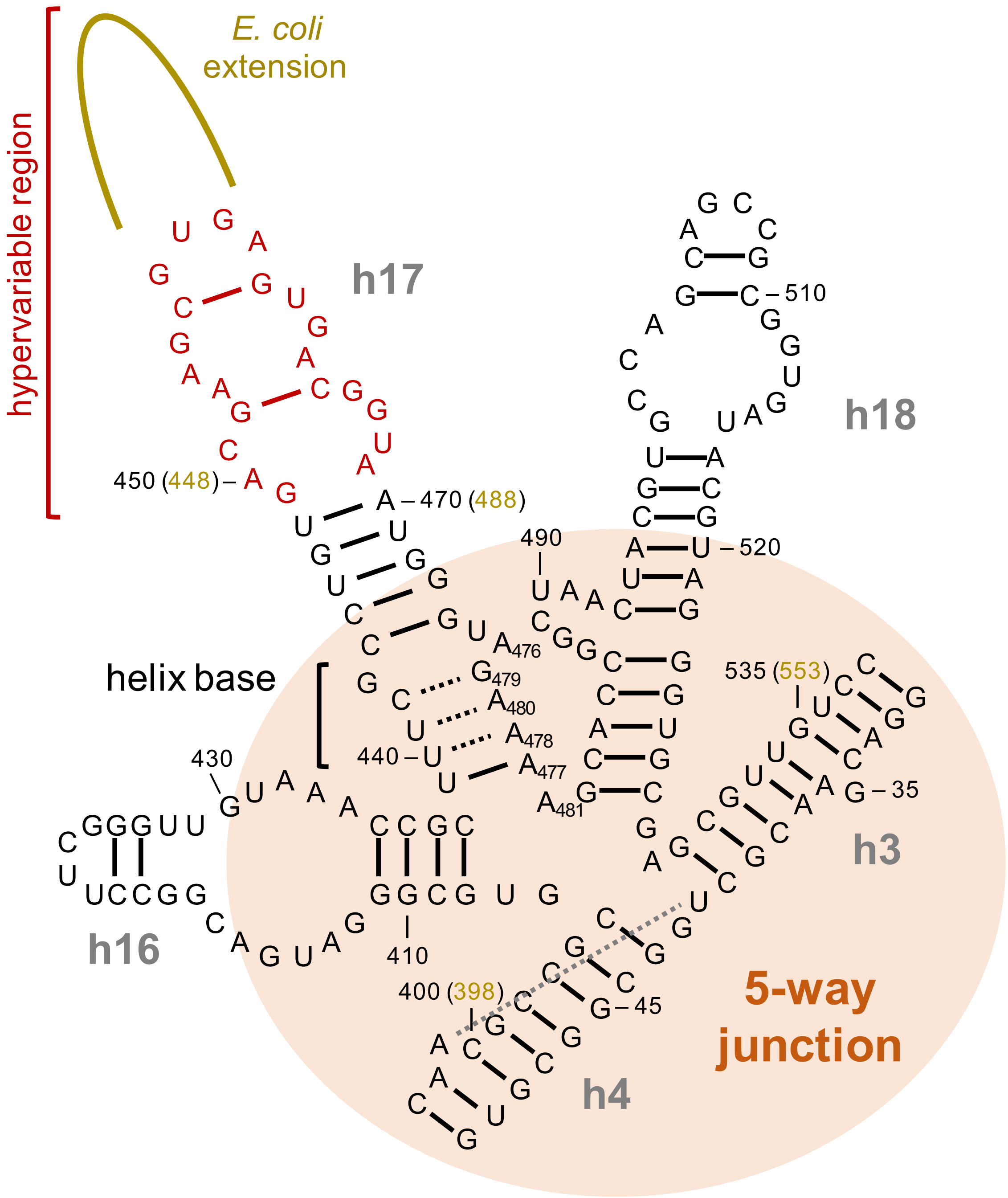

Figure S12. The 5-way junction in *C. acnes* 16S rRNA. Helices h3, h4, h16, h17 and h18 form the 5-way junction (pink circle). Helices h16 and h17 are a part of the variable region 3 (V3) of the 16S rRNA (nucleotides 328 (326) - 483 (501)). Position of *E. coli* extension (yellow) in hypervariable region of h17 is shown. Changes in size in that region do not affect the base of the h17 and the structure of the 5-way junction, which is crucial for the 30S ribosomal subunit assembly.

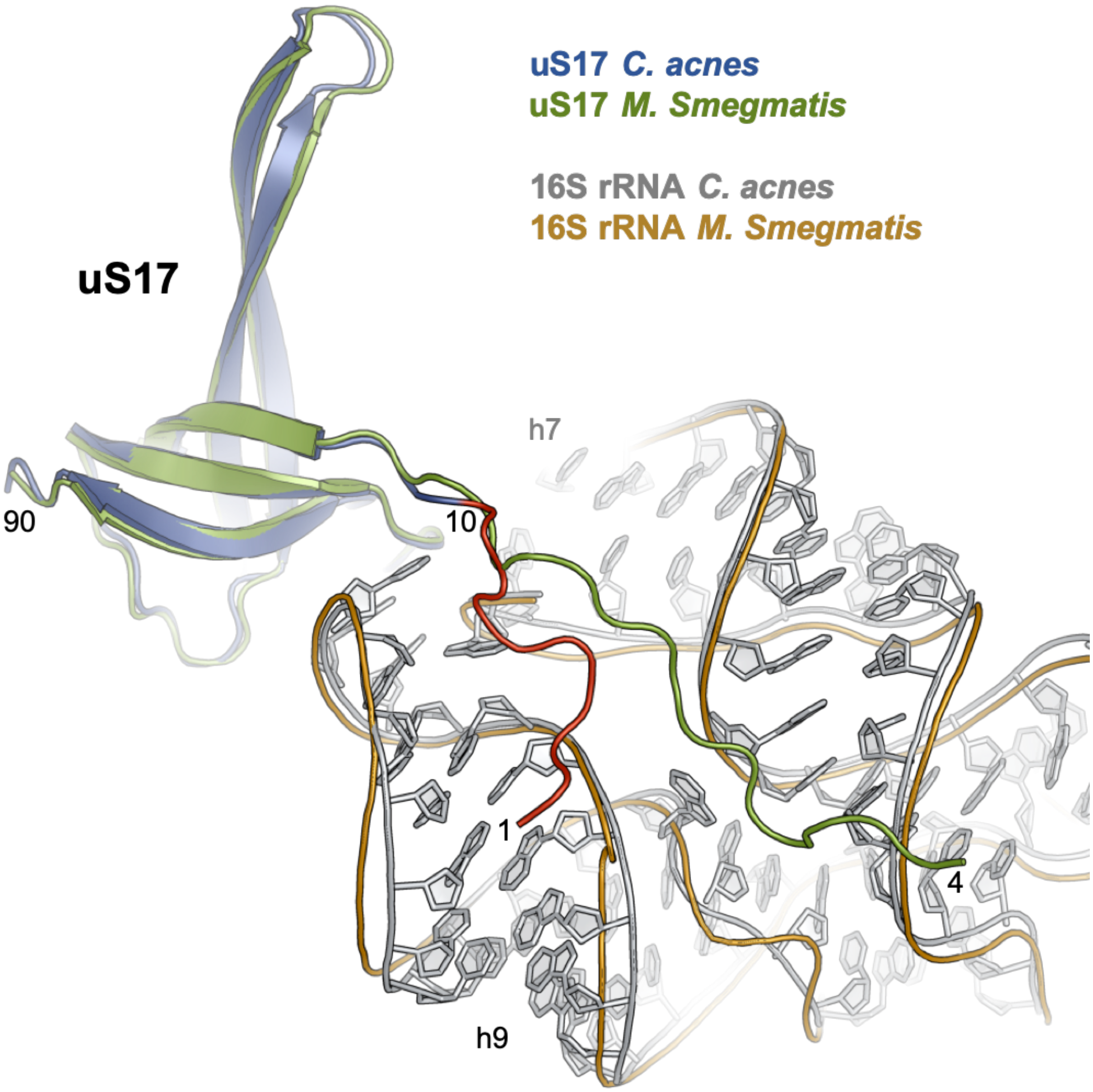

Figure S13. N terminus of uS17 from *C. acnes* interacts with the major groove of h9. Its conserved domain is in blue and *C. acnes*-specific extension is in red. Structure of *C. acnes* 70S subunit (gray) was superimposed with that of the *M. smegmatis* (PDB ID 5O61, gold). uS17 from *M. smegmatis* is shown in green. Some amino acid sequence numbers are shown as reference markers.

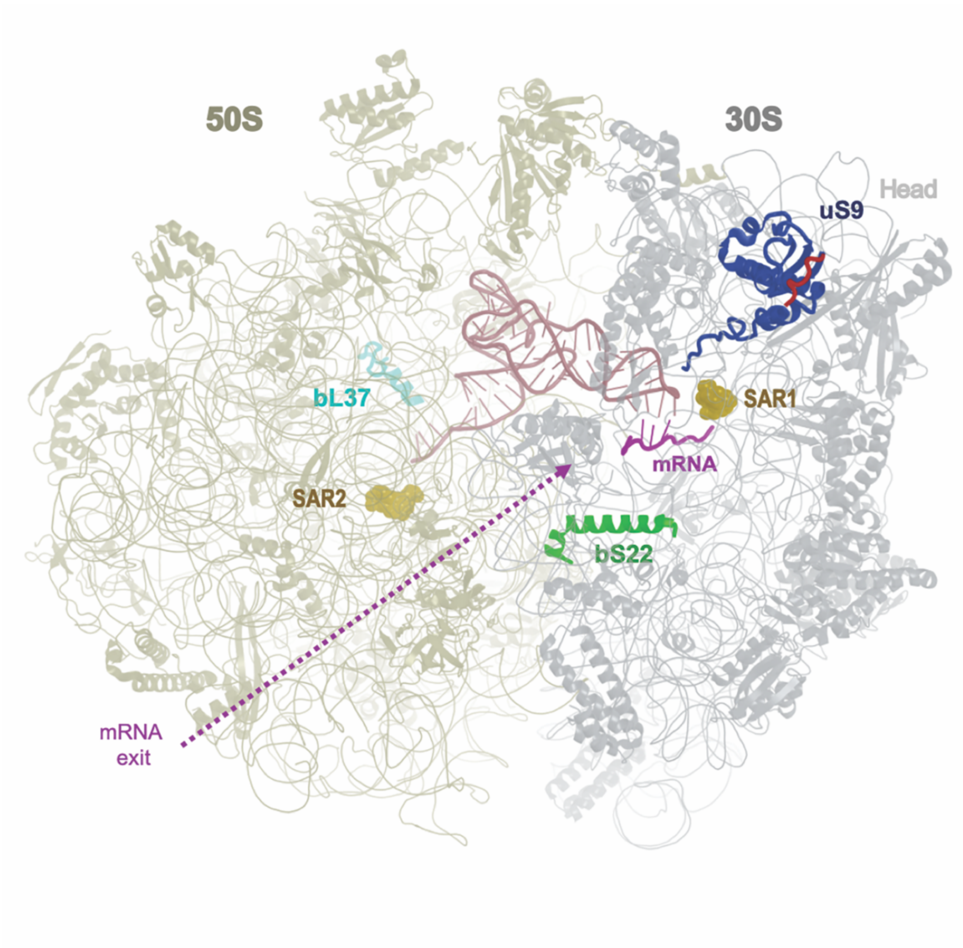

Figure S14. uS9 is located above the mRNA exit site on the 30S ribosomal subunit. uS9 conserved domain is in blue and *C. acnes*-specific extension is in red.

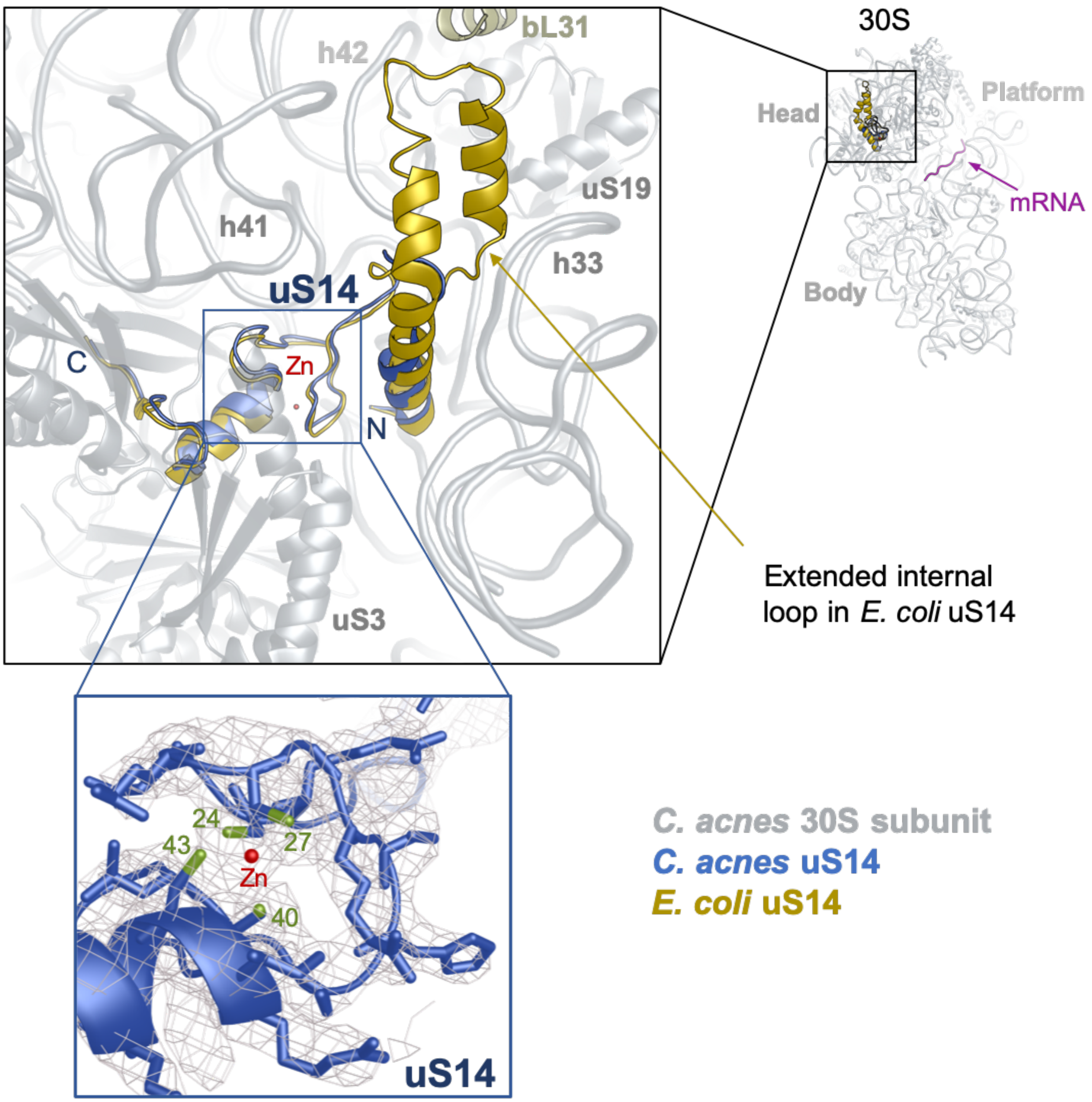

Figure S15. Ribosomal protein uS14 in the *C. acnes* 30S subunit. We observed Zn^{2+} binding variant of uS14 (blue). Superposed uS14 from *E. coli* (yellow) does not have the Zn^{2+} binding motif. The 41 amino acids long internal loop of *E. coli* uS14 reaches to the C-terminus of bL31, interacts with uS19 and helices h33, h41, h42 of the head domain of the 30S subunit. Top right panel shows position of the uS14 in the head domain of the small ribosomal subunit (30S). Bottom panel shows the Zn^{2+} binding site and the corresponding local EM density map (in mesh). Cysteine amino acid residues (green), forming the Zn^{2+} (red) binding site, and their positions in *C. acnes* uS14 are shown.

```
C. acnes, Zn2+  1  MAKTALKVKAARKPKF----------------------------------------G  17
C. acnes, Zn-   1  MAKKSKIVAQKKREKLVAQYAERRAELKAIMKCPTASLDERMEASRKLSRLPRDSSP  57
E. coli,  Zn-   1  MAKQSMKAREVKRVALADKYFAKRAELKAIISDVNASDEDRWNAVLKLQTLPRDSSP  57

C. acnes, Zn2+ 18  VRAYTRCQRCGRPHSVYRKFGLCRICLREMAHAGQLPGVTKSSW   61
C. acnes, Zn-  58  VRLRNRDQVDGRPRGYVGKAGVSRARFREMAHRGELPGITKSSW  101
E. coli,  Zn-  58  SRQRNRCRQTGRPHGFLRKFGLSRIKVREAAMRGEIPGLKKASW  101
```

Figure S16. uS14 amino acid sequences alignment. Sequences were aligned using Clustal O (1.2.4) software (https://www.ebi.ac.uk/Tools/msa/clustalo/) and manually refined based on superposition of *E. coli* (PDB ID 7K00) and *C. acnes* 70S ribosomes. Identical amino acids for all three proteins are highlighted in blue, for Zn^- proteins are in bold. Four cysteine amino acid residues forming $Zn2^+$ binding site are in red. The sequence corresponding to the extended internal loop in *E. coli* uS14 is highlighted in yellow. Accession numbers: *C. acnes* - A0A2B7JMX1 (UniProt, $Zn2^+$), BCB14138.1 (GenBank, Zn^-), *E. coli* - (PDB ID 7K00, ref. 19).

```
C. acnes        -UUUCCGGUGGCCAUAGUGGAAGGGAAACACCCGGUCCCAUUCCGAACCCGGUCGUUAAG   59
E. coli         -UGCCUGGCGGCCUUAGCGCGGUGGUCCCACCUGACCCCAUGCCGAACUCAGAAGUGAAA   59
M. smegmatis    GUUACGGCGGUCCAUAGCGGCAGGGAAACGCCCGGUCCCAUCCCGAACCCGGAAGCUAAG   60
                 *  * *  * ** *** *    **   * ** *  ***** ****** * *  ** **

C. acnes        CCUUCCAACGCUGAUGGUACUGCACGAGGGAUCGUGUGGGAGAGUAAGACGCUGCCGGAAUAC 122
E. coli         CGCCGUAGCGCCGAUGGUAGUGUGGG-GUCUCCCCAUGCGAGAGUAGGGAACUGCCAGGCAU  120
M. smegmatis    CCUGCCAGCGCCGAUGAUACUACCCAUC----CGGGUGGAAAAGUAGGACACCGCCGAACAC  118
                *     * *** **** ** *           *   **  * **** *   * ***
```

Figure S17. 5S rRNA nucleotide sequences alignment. Nucleotide sequences of the 5S rRNA were aligned using Clustal O (1.2.4) software (https://www.ebi.ac.uk/Tools/msa/clustalo/). Accession numbers: *C. acnes* - NR_075172, *E. coli* - NR_075284.1, *M. smegmatis* - PDB ID 5O61, ref. 7.

Table S1. Cryo-EM data collection, refinement and validation statistics

	70S-SAR-mRNA-tRNA ribosomal complex (EMDB-26959) (PDB 8CRX)
**Data collection and processing**	
Magnification	81,000
Voltage (kV)	300
Electron exposure (e–/Å^2)	30.56
Defocus range (μm)	0.5-2
Pixel size (Å)	1.068
Symmetry imposed	C1
Initial particle images (no.)	1,108,852
Final particle images (no.)	70,853
Map resolution (Å)	2.78
FSC threshold	0.143
Map resolution range (Å)	2.2-6.2
**Refinement**	
Initial model used (PDB code)	7K00 and 5O61
Map sharpening *B* factor (Å^2)	-27.9
Model composition	
Non-hydrogen atoms	144602
Protein residues	5733
Ligands	413
R.m.s. deviations	
Bond lengths (Å)	0.004 (1)
Bond angles (°)	0.540 (39)
Validation	
MolProbity score	1.85
Clashscore	8.24
Ramachandran plot	
Favored (%)	94.00
Allowed (%)	5.58
Disallowed (%)	0.43

Table S2. *Cutibacterium acnes* ribosomal proteins and RNAs

Name	Chain ID	Length (residues)	Modeled residues	UniProt ID (proteins)	Notes
		ca/ms/ec*			
**30S (small subunit)**					
bS1		498/479/557		A0A533IL68	not visible
uS2	B	283/277/241	1-233	A0A2B7I756	
uS3	G	269/275/233	2-207	A0A2B7I5Y3	
uS4	D	201/201/206	2-201	A0A2B7JMY9	
uS5	E	215/214/167	32-210	A0A2B7I5L8	
bS6	F	96/96/135	1-96	A0A2C6L3A1	
uS7	I	156/156/179	2-156	A0A2B7ITZ4	
uS8	H	135/132/130	2-135	A0A2B7ITQ7	
uS9	J	173/150/130	40-173	A0A2B7JN42	
uS10	M	103/101/103	5-102	A0A085B5E4	
uS11	K	135/138/129	19-135	A0A2B7I645	
uS12	L	123/124/124	2-123	A0A2C6LJN0	
uS13	N	123/124/118	2-123	A0A2C6LKT6	
uS14	S	61/61/101	2-61	A0A2B7JMX1	Zn^{2+} binding
uS15	O	87/89/89	1-87	A0A2B7I743	
bS16	P	147/156/82	2-129	A0A2B7IXM7	
uS17	Q	90/98/84	1-90	A0A2B7JMS7	
bS18	R	79/84/75	9-75	A0A2C6LI71	
uS19	U	93/93/92	2-85	A0A2B7ITR7	
bS20	T	88/86/87	2-88	A0A2B7I592	
bS21	-	n.a./**/71	-	n.a.**	not present**
bS22	X	33/33/n.a.	2-33	A0A533INE0	
16S rRNA	A	1537/1528/1542	6-1521	CP012350.1 *** region 607197-608733	not built: 83-89 1014-1021
**50S (large subunit)**					
uL1	-	235/235/234	-	A0A2B7I2W2	not visible
uL2	c	278/278/273	2-275	A0A2B7JMV5	
uL3	d	223/217/209	4-217	A0A085B5E3	
uL4	e	301/215/201	3-212	A0A085B5E2	
uL5	f	210/187/179	7-190	A0A3E2DDM1	
uL6	g	180/179/177	2-178	A0A2B7I5S3	
bL9	-	149/151/149	-	A0A371N5J5	not visible
uL10	-	204/175/165	-	A0A2B7I357	not visible
uL11	-	143/142/142	-	A0A533IKS4	not visible
uL13	i	147/147/142	2-147	A0A2B7I6I7	
uL14	j	122/122/123	1-122	A0A2B7JMZ8	
uL15	k	146/147/144	2-145	A0A2B7IUA0	
uL16	l	139/139/136	1-136	A0A2B7I5M8	
bL17	m	187/199/127	2-121	A0A085AZY3	
uL18	n	127/127/117	2-127	A0A2B7JN02	
bL19	o	117/113/115	2-115	A0A2C6LKT3	
bL20	p	123/129/118	2-120	A0A2B7I6V3	
bL21	q	102/103/103	1-102	A0A085B4N9	
uL22	r	153/153/110	1-132	A0A2B7ITY5	
uL23	s	102/100/100	3-97	A0A2C6LEQ5	
uL24	t	122/105/104	1-120	A0A085B5D2	48-60 not built
bL25	u	205/215/94	3-181	A0A2B7IYF8	

bL27	v	89/88/85	7-84	A0A2B7IGT1	
bL28	w	61/64/78	2-61	A0A2B7I7B7	Zn^{2+} binding
uL29	x	77/77/63	3-71	A0A2B7JB12	
uL30	y	60/61/59	1-58	A0A2B7I5Y4	
bL31	4	69/75/70	1-66 C	A0A2B7JUW7	Zn^{2+} binding
bL32	z	63/57/57	2-63	A0A2B7I771	Zn^{2+} binding
bL33	0	56/55/55	7-56	A0A2C6LJM0	Zn^{2+} binding
bL34	1	44/47/46	1-44	A0A2B7IDI8	
bL35	2	68/64/65	2-68	A0A085AZG2	
bL36	3	37/37/38	1-37	A0A2B7JN22	Zn^{2+} binding
bL37	V	24/24/n.a.	2-24	KEY34928.1***	
23S rRNA	a	3086/3120/2904	2-3080	CP012350 *** region: 608942..612027	not built: 292-300 1494-1592 2292-2359
5S rRNA	b	120/118/120	1-120	CP012350 *** region: 612130..612249	

*) ca - *Cutibacterium acnes,* ms - *Mycobacterium smegmatis,* ec - *Escherichia coli*
**) No reference sequence for bS21 of *C. acnes* is deposited in the NCBI and UniProt databases
***) GenBank ID

Table S3. Relative minimum inhibitory concentration of SAR

**Gram-positive bacteria**	MIC*	Base pair** (1782:2586)	HIS69*** (uL4)
Cutibacterium acnes	+	C:C	G
Staphylococcus aureus	+	C:C	G
Staphylococcus haemolyticus	+	C:C	G
Streptococcus pyogenes	+	C:C	G
Streptococcus agalactiae	++	C:C	G
Enterococcus faecalis	+++	C:C	G
Enterococcus faecium	++	C:C	G
Mycolicibacterium smegmatis	NA****	U:U	G
Mycobacterium tuberculosis	NA	U:U	G
**Gram-negative bacteria**			
Escherichia coli	+++	U:U	G
Klebsiella pneumoniae	+++	U:U	G
Salmonella typhimurium	+++	U:U	G
Morganella morganii	+++	U:U	G
Proteus mirabilis	+++	U:U	G
Providencia stuartii	+++	U:U	G
Serratia marcescens	+++	U:U	G
Thermus thermophilus	NA	C:C	H

*) MICs were reported in:
Zhanel, G., Critchley, I., Lin, L.Y. and Alvandi, N. (2019) Microbiological Profile of Sarecycline, a Novel Targeted Spectrum Tetracycline for the Treatment of Acne Vulgaris. Antimicrobial agents and chemotherapy, 63.
Bunick, C.G., Keri, J., Tanaka, S.K., Furey, N., Damiani, G., Johnson, J.L. and Grada, A. (2021) Antibacterial Mechanisms and Efficacy of Sarecycline in Animal Models of Infection and Inflammation. Antibiotics (Basel), 10.
**) 23S rRNA, *E. coli* numbering
***) Amino acid residue in the position corresponding to HIS69 in uL4 of *T. thermophilus*
****) Not available
